# *Spirulina platensis* Suppressed iNOS and Proinflammatory Cytokines in Lipopolysaccharide-Induced BV2 Microglia

**DOI:** 10.3390/metabo12111147

**Published:** 2022-11-20

**Authors:** Ee-Ling Ngu, Cheng-Yau Tan, Nicole Jean-Yean Lai, Kah-Hui Wong, Siew-Huah Lim, Long Chiau Ming, Kuan-Onn Tan, Siew-Moi Phang, Yoon-Yen Yow

**Affiliations:** 1 Department of Biological Sciences, School of Medical and Life Sciences, Sunway University, Bandar Sunway 47500, Malaysia; 2 Department of Anatomy, Faculty of Medicine, Universiti Malaya, Kuala Lumpur 50603, Malaysia; 3 Department of Chemistry, Faculty of Science, Universiti Malaya, Kuala Lumpur 50603, Malaysia; 4 PAPRSB Institute of Health Sciences, Universiti Brunei Darussalam, Gadong BE1410, Brunei; 5 Faculty of Applied Sciences, UCSI University, Kuala Lumpur 56000, Malaysia; 6 Institute of Ocean and Earth Sciences (IOES), Universiti Malaya, Kuala Lumpur 50603, Malaysia

**Keywords:** *Spirulina platensis*, neuroprotective, anti-neuroinflammation, antioxidants, nitric oxide, BV2 microglia

## Abstract

The disease burden of neurodegenerative diseases is on the rise due to the aging population, and neuroinflammation is one of the underlying causes. *Spirulina platensis* is a well-known superfood with numerous reported bioactivities. However, the effect of *S. platensis* Universiti Malaya Algae Culture Collection 159 (UMACC 159) (a strain isolated from Israel) on proinflammatory mediators and cytokines remains unknown. In this study, we aimed to determine the anti-neuroinflammatory activity of *S. platensis* extracts and identify the potential bioactive compounds. *S. platensis* extracts (hexane, ethyl acetate, ethanol, and aqueous) were screened for phytochemical content and antioxidant activity. Ethanol extract was studied for its effect on proinflammatory mediators and cytokines in lipopolysaccharide (LPS)-induced BV2 microglia. The potential bioactive compounds were identified using liquid chromatography-mass spectrometric (LC-MS) analysis. Ethanol extract had the highest flavonoid content and antioxidant and nitric oxide (NO) inhibitory activity. Ethanol extract completely inhibited the production of NO via the downregulation of inducible NO synthase (iNOS) and significantly reduced the production of tumor necrosis factor (TNF)-α and interleukin (IL)-6. Emmotin A, palmitic amide, and 1-monopalmitin, which might play an important role in cell signaling, have been identified. In conclusion, *S. platensis* ethanol extract inhibited neuroinflammation through the downregulation of NO, TNF-α and IL-6. This preliminary study provided insight into compound(s) isolation, which could contribute to the development of precision nutrition for disease management.

## 1. Introduction

The global life expectancy was reported to increase to 73 years in 2017, accompanied by an increase in age-related disease burdens, including neurodegenerative diseases. As the major neurodegenerative disease, Alzheimer’s disease (AD) has contributed to a 38.3% increment in disability-adjusted life-years (DALYs) within 10 years [1]. In addition, the number of death caused by AD has increased from 1.004 million in 2010 to 1.639 million in 2019, making AD the sixth leading cause of global deaths [2]. Neurodegenerative diseases are characterized by gradual neuronal loss due to brain injuries and pathological aging, which exaggerates age-related cognitive decline [3]. Neuroinflammation is one of our body’s defense mechanisms, which maintains body homeostasis and protects the central nervous system (CNS) against pathogenic insults [4]. As the primary mediator of neuroinflammation, microglia undergo activation upon detection of stimuli such as infectious agents, damaged cells, and proinflammatory mediators in the brain [5,6]. Dysregulated neuroinflammation is an abnormal condition that occurs when persistent stimuli or failure in the resolution mechanism triggers uncontrolled microglia activation [4,7]. Uncontrolled activation subsequently results in the overproduction of reactive oxygen species (ROS), proinflammatory mediators (nitric oxide (NO) and prostaglandin E_2_ (PGE_2_)), and cytokines (interleukin-6 (IL-6) and tumor necrosis factor-α (TNF-α)) that gradually damage the neuronal cell [8,9,10].

The Food and Drug Administration (FDA) -approved drugs for the treatment of AD are mainly neurotransmitter regulators, where donepezil, galantamine, and rivastigmine are cholinesterase inhibitors (ChEIs) that prevent the breakdown of a neurotransmitter called acetylcholine (ACh), and memantine is an N-methyl-D-aspartic acid (NMDA) receptor antagonist that prevents the overproduction of another neurotransmitter, glutamate [11]. However, these drugs are symptomatic treatments and are accompanied by adverse effects, such as nausea, dizziness, and constipation [12]. Hence, there is an urgent need to discover novel natural bioactive compounds with anti-neuroinflammatory activity and minimal side effects. In the past decade, marine algae have shown promising neuroprotective properties in preclinical and clinical studies [13]. For instance, the red macroalga *Gracilaria manilaensis* has been reported with antioxidant, neuritogenic and anti-cholinesterase activities [14,15]. In 2019, sodium oligomannate (GV-971), an oligosaccharide derived from brown algae, became the first AD drug approved by FDA since 2003 [16]. GV-971 ameliorated the progression of AD by inhibiting gut dysbiosis-promoted neuroinflammation in an AD mouse model [17]. This breakthrough highlighted the therapeutic potential of marine algae for neurodegenerative diseases. *Spirulina platensis* (*Arthrospira platensis*) is a blue-green microalga (cyanobacterium) with high content of proteins, vitamins, pigments, fatty acids, and minerals [18]. Various therapeutic activities have been reported in *S. platensis*, such as immunomodulatory [19], anticancer [20], antimicrobial [21,22], antioxidant [23], and anti-inflammatory [24,25] activities. Antioxidant and anti-inflammatory activities of *S. platensis* protected dopaminergic neurons in rat models of AD and Parkinson’s disease (PD) [26,27]. Furthermore, *S. platensis* promoted neurite outgrowth in PC-12Adh cells [28] and spinal cord injury recovery in rats [29]. However, there is a lack of evidence for the neuroprotective activity of *S. platensis* via the regulation of neuroinflammation in microglia.

BV2 microglia is an immortalized murine microglia cell line that shows similar gene expression as primary microglia upon lipopolysaccharide (LPS) stimulation [30]. LPS produced by gram-negative bacteria promoted the production of proinflammatory cytokines by microglia, which in turn damage the neuronal cell [31,32]. Therefore, the present study aimed to determine the anti-neuroinflammatory activity of *S. platensis* UMACC 159 culture strain extracts in LPS-induced BV2 microglia and to identify the potential bioactive compound(s).

## 2. Materials and Methods

### 2.1. Chemicals and Reagents

Gallic acid (Shanghai, China), quercetin (Bangalore, India), aluminum chloride, 2,2-diphenyl-1-picrylhydrazyl (DPPH) (Munich, Germany), ascorbic acid (Tokyo, Japan), Minimum Essential Medium Eagle (MEM; M3024), sodium bicarbonate, fetal bovine serum (FBS), penicillin-streptomycin, LPS from *Escherichia coli* O55:B5, and N(γ)-nitro-L-arginine methyl ester (L-NAME) (St. Louis, Mo, USA) were purchased from Sigma. The Folin Ciocalteu reagent and 3-(4,5-dimethylthiazol-2-yl)-2,5-diphenyltetrazolium bromide (MTT) were purchased from Merck KGaA (Darmstadt, Germany) and Merck & Co. (Rahway, NJ, USA) respectively. The 2,2′-Azino-bis(3-ethylbenzothiazoline-6-sulfonic acid (ABTS) and phenylmethylsulfonyl fluoride (PMSF) were purchased from Roche diagnostics (Mannheim, Baden-Wurttemberg, Germany); the ELISA kits were purchased from R&D Systems (Minneapolis, MN, USA); protease inhibitor (A32865) and Pierce bicinchoninic acid assay (BCA) protein assay kit were purchased from Thermo Fisher Scientific (Waltham, MA, USA); horseradish peroxidase (HRP)-conjugated goat anti-rabbit secondary antibody was purchased from Invitrogen (Rockford, IL, USA); and WesternBright enhanced chemiluminescence (ECL) spray was purchased from Advansta (San Jose, CA, USA). The Griess reagent nitrite measurement kit, cell lysis buffer (9803), and primary antibodies (iNOS (#13120); COX-2 (#12282), and β-actin (#4970)) were purchased from Cell Signaling Technology (Danvers, MA, USA).

### 2.2. Solvent Extracts Preparation

**Figure 1:**
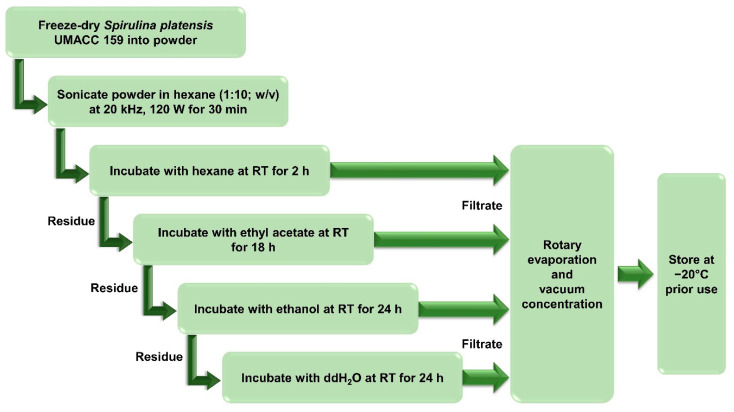
Preparation of *S. platensis* extracts using ultrasound-assisted extraction (UAE) and sequential extraction (SE). RT: room temperature; ddH_2_O: double distilled water.

*S. platensis* UMACC 159 culture strain was obtained from the University of Malaya Algae Culture Collection (UMACC). The culture strain was identified and authenticated by experts in the Algae Research Laboratory at Universiti Malaya. *S. platensis* was freeze-dried (LaboGene, Brigachtal, Germany) and stored at −20 °C prior to use. Solvent extracts were prepared using ultrasound-assisted extraction (UAE) and sequential extraction (SE). Five g of *S. platensis* were immersed in hexane and sonicated at 20 kHz, 120 W, for 30 min. The sample was consecutively incubated in solvents with increasing polarity at the ratio of 1:10 (*w*/*v*) for the indicated incubation time (Figure 1**)**. After each incubation, the extract was filtered and dried using a rotary evaporator (Fisher Scientific EYELA N-1200A, Tokyo, Japan) and vacuum concentrator (LaboGene, Brigachtal, Germany). Dried extracts were stored at −20 °C prior to use. The yield (%) of the extract was calculated using Formula (1).

(1) $$\mathrm{Yield}\;\left(\%\right)=\frac{\mathrm{Dried}\;\mathrm{mass}\;\mathrm{of}\;\mathrm{extract}\;\left(g\right)}{\mathrm{Initial}\;\mathrm{weight}\;\mathrm{of}\;\mathrm{powder}\;\left(g\right)}\times 100\%$$

### 2.3. Phytochemical Screening

#### 2.3.1. Total Phenolic Content (TPC)

TPC was quantified according to Pang et al. [14]. Briefly, 5 µL samples diluted with 350 µL ddH_2_O were incubated with 25 µL Folin Ciocalteu reagent in the dark for 4 min. The mixture was further diluted with 45 µL ddH_2_O and incubated with 75 µL of 20% sodium carbonate in the dark for 1 h. Absorbance was measured at 750 nm using the UV-Vis spectrophotometer microplate reader (Infinite 200 Pro, Mannedorf, Switzerland). TPC was calculated with gallic acid as standard and expressed as mg of gallic acid equivalent (GAE) per g of extract (mg GAE/g).

#### 2.3.2. Total Flavonoid Content (TFC)

TFC was quantified according to Pang et al. [14]. Briefly, 10 µL sample diluted with 490 µL ddH_2_O were incubated with 250 µL of 2% methanolic aluminum chloride and 250 µL of 1 M sodium acetic acid in the dark for 15 min. Absorbance was measured at 425 nm. TFC was calculated with quercetin as standard and expressed as mg of quercetin equivalent (QE) per g of extract (mg QE/g).

### 2.4. Antioxidant Capacity

#### 2.4.1. ABTS Scavenging Activity

Scavenging activity on ABTS radical was determined according to Pang et al. [14]. Briefly, 7 mM ABTS was activated with 2.45 mM potassium persulfate in the dark for 16 h. Activated ABTS was diluted with ethanol to achieve an absorbance of 0.7 ± 0.02. Next, a 0.1 mL sample was incubated with 1 mL diluted ABTS in the dark for 6 min. Absorbance was measured at 734 nm. Scavenging activity was calculated according to Formula (2) and expressed as half-maximum effective concentration (EC_50_) at which the radicals were scavenged by half. Ascorbic acid served as the positive control.

(2) $$\mathrm{ABTS}\;\mathrm{scavenging}\;\mathrm{activity}\;\left(\%\right)=1-\frac{\mathrm{Absorbance}\;\mathrm{of}\;\mathrm{samples}}{\mathrm{Initial}\;\mathrm{absorbance}\;\mathrm{of}\;\mathrm{ABTS}}\times 100\%$$

#### 2.4.2. DPPH Scavenging Activity

Scavenging activity on DPPH radical was determined according to Pang et al. [14]. Briefly, a 50 µL sample was incubated with 1 mL of 0.1 mM DPPH in the dark for 30 min. Absorbance was measured at 518 nm. Scavenging activity was calculated according to Formula (3) and expressed as EC_50_. Ascorbic acid served as the positive control.

(3) $$DPPH\;scavenging\;activity\;\left(\%\right)=1-\frac{Absorbance\;of\;samples}{Absorbance\;of\;sample\;at\;0\;mg/mL}\times 100\%\;$$

#### 2.4.3. Reducing Power

The reducing power was determined according to Pang et al. [14]. Briefly, 100 µL samples were incubated with 250 µL of 0.2 M phosphate buffer and 250 µL of 1% potassium ferricyanide at 50 °C for 20 min. The mixture was added with 250 µL of 10% trichloroacetic acid and centrifuged at 3000 rpm for 10 min. 250 µL of the solution was mixed with 250 µL ddH_2_O and 250 µL of 0.1% iron (III) chloride. Absorbance was measured at 700 nm. Reducing power was expressed as EC_50_, with ascorbic acid serving as the positive control.

### 2.5. Cell Culture

BV2 microglia (EP-CL-0493) was purchased from Elabscience. The cells were cultured in MEM supplemented with 2.2 g sodium bicarbonate, 10% FBS, and 1% penicillin-streptomycin in a 5% CO_2_-humidified incubator at 37 ± 2 °C.

### 2.6. Cell Viability

BV2 microglia were seeded at a cell density of 62.5 × 10^3^ cells/well in a 96-well plate overnight. The cells were treated with extracts dissolved in fresh medium for 24 h, followed by incubation with 0.5 mg/mL MTT for 4 h. The culture medium was replaced with 100 µL DMSO to dissolve the formazan in viable cells. Absorbance was measured at 570 nm with 630 nm as the reference wavelength. Cell viability was calculated using Formula (4). Cells incubated in a medium only served as the negative control.

(4) $$\mathrm{Cell}\;\mathrm{viability}\;\left(\%\right)=\frac{\mathrm{Absorbance}\;\mathrm{of}\;\mathrm{sample}}{\mathrm{Absorbance}\;\mathrm{of}\;\mathrm{negative}\;\mathrm{control}}\times 100\%$$

### 2.7. Anti-Neuroinflammatory Activity

#### 2.7.1. Griess Assay

Inhibitory activity on the production of NO was determined by measuring the amount of nitrite, a stable oxidation product of NO, in the culture medium using the Griess reagent nitrite measurement kit. BV2 microglia were seeded at a cell density of 62.5 × 10^3^ cells/well in a 96-well plate overnight. The cells were pre-treated with a fresh medium containing a selected concentration of extracts or 250 μM L-NAME for 2 h, followed by incubation with 1 µg/mL LPS for 24 h. The culture medium was mixed with an equal volume of Griess reagent, and the absorbance was measured at 550 nm. The amount of NO (µM) was calculated with sodium nitrite as the standard. To justify the inhibitory effect on NO production by the extracts, the cell viability of the treated cells was tested using MTT, as mentioned above (Experiment 2.6). NO production (%) was calculated using Formula (5). Cells incubated with medium only served as the negative control; LPS only served as the LPS control; L-NAME and LPS served as the positive control. Extract with the highest NO inhibitory activity was chosen for the subsequent assays.

(5) $$\mathrm{NO}\;\mathrm{production}\;\left(\%\right)=\frac{\mathrm{Amount}\;\mathrm{of}\;\mathrm{NO}\;\mathrm{in}\;\mathrm{sample}}{\mathrm{Amount}\;\mathrm{of}\;\mathrm{NO}\;\mathrm{in}\;\mathrm{LPS}\;\mathrm{control}}\times 100\%$$

#### 2.7.2. Enzyme-Linked Immunosorbent Assay (ELISA)

BV2 microglia were seeded at a cell density of 625 x 10^3^ cells/well in a 6-well plate overnight. The cells were pre-treated with a fresh medium containing a selected concentration of ethanol extract for 2 h, followed by incubation with 1 µg/mL LPS for 24 h. The amount of PGE_2_, TNF-α, and IL-6 in the culture medium was measured using the ELISA kit according to the manufacturer’s instructions. Briefly, the culture medium (with the addition of mouse anti-PGE_2_ detection antibody for PGE_2_) was incubated in the well pre-coated with the respective capture antibody for the indicated incubation period. The wells were washed (only for TNF-α and IL-6) and further incubated with HRP-conjugated antibody or PGE_2_ for 2 h. The wells were washed and incubated with a substrate solution in the dark for 30 min. A stop solution was added, and absorbance was measured at 450 nm with 570 nm as the reference wavelength. The inhibition (%) was calculated using Formula (6). Cells incubated with medium only served as the negative control; LPS only served as the LPS control.

(6) $$\mathrm{Inhibition}\;\left(\%\right)=\frac{\mathrm{Amount}\;\mathrm{in}\;\mathrm{LPS}\;\mathrm{control}\;-\;\mathrm{Amount}\;\mathrm{in}\;\mathrm{sample}}{\mathrm{Amount}\;\mathrm{in}\;\mathrm{LPS}\;\mathrm{control}}\times 100\%$$

#### 2.7.3. Western Blot Analysis

BV2 microglia were seeded at a cell density of 625 × 10^3^ cells/well in a 6-well plate overnight. The cells were treated as mentioned in Experiment 2.7.2. Cells were washed with ice-cold phosphate-buffered saline (PBS) and lysed with cell lysis buffer containing PMSF and protease inhibitor on ice for 5 min. Cell lysates were collected and centrifuged at 150,000 rpm at 4 °C for 10 min to remove the cell debris. Protein concentration was quantified using the BCA protein assay kit. An equal amount of protein was separated using 8% sodium dodecyl sulfate-polyacrylamide gel electrophoresis (SDS-PAGE) and electrophoretically transferred onto a nitrocellulose membrane. The membrane was blocked with 5% nonfat milk for 1 h and subsequently incubated with the primary antibody (COX-2 (1:1000), iNOS (1:500), or β-actin (1:1000)) at 4 °C overnight. The membrane was washed and incubated with HRP-conjugated goat anti-rabbit secondary antibody (1:10,000) for 1 h. After washing, the membrane was added with ECL, and the signal was visualized using the G:BOX Chemi XX9 gel doc system and GeneSys image acquisition software (Syngene, Cambridge, UK). Band intensity was quantified using Image J software (version 1.50, Wayne Rasband, National Institutes of Health, Bethesda, MD, USA). The original Western blot images were shown in Figure S1.

### 2.8. Bioactive Compounds Identification

The ethanol extract was sent for liquid chromatography-mass spectrometric (LC-MS) analysis at Monash University (Malaysia). Compounds separation was performed on the Agilent 1290 Infinity LC system coupled to Agilent 6520 Accurate-Mass quadrupole time-of-flight (Q-TOF) mass spectrometer with dual electrospray ionization (ESI) source (Agilent Technologies, Santa Clara, CA, USA), operated in the positive-ion mode. The ethanol extract was loaded into a Narrow-Bore 2.1 × 150 mm, 3.5 μm particle size Agilent Zorbax Eclipse XDB-C18 column (P/N: 930990-902). Separation was performed using solvent A (0.1% formic acid in water) and solvent B (0.1% formic acid in acetonitrile) with the gradient setting of 5% B at the 0 and 5 min, followed by 100% B at the 20 min and 25 min. The total run time was 30 min (including 5 min post-run time) at a flow rate of 0.5 mL/min. Autosampler temperature was maintained at 4 °C with an injection volume of 1 μL, while column temperature was set at 25 °C. The settings of the mass spectrometer included: capillary voltage: 4000 V, fragmentor voltage: 125 V, skimmer: 65 V, and liquid nebulizer: 45 psig. The drying gas temperature was maintained at 300 °C at a flow rate of 10 L/min. The acquisition rate was 1.03 spectra/sec, and the mass spectrum was scanned from *m/z* 100 to 3200.

The data were processed using Agilent MassHunter Qualitative Analysis B.07.00 software (Agilent Technologies, Santa Clara, CA, USA) with the Molecular Feature Extraction (MFE) small molecule algorithm. Compounds were identified using the Molecular Formula Generator (MFG) software (Agilent Technologies, Santa Clara, CA, USA) and through matching with the Metlin_AM_PCDL-N-170502.cdb database.

### 2.9. Statistical Analysis

All experiments were repeated three times. All data were expressed as mean ± standard error (SE) and statistically analyzed using 1-way ANOVA with post hoc testing (GraphPad Prism ver. 5.02, Dotmatics, San Diego, CA, USA). Values of *p* ≤ 0.05 were considered to have a significant difference.

## 3. Results

### 3.1. Yield Percentage and Phytochemical Content

The aqueous extract had the highest yield (27.75 ± 0.877%), and the yield was significantly (*p* ≤ 0.05) higher than ethyl acetate, ethanol, and hexane extracts by approximately 5.23-, 8.04-, and 9.31-fold, respectively. Based on Table 1, all solvent extracts showed positive results for TPC, and the amount increased as solvent polarity increased from hexane to aqueous. Notably, aqueous, ethanol, and ethyl acetate extracts showed positive results for TFC but not for hexane extract. In contrast with TPC, TFCs were not affected by solvent polarity, as ethanol extract had the highest content (83.41 ± 2.049 mg QE/g) while ethyl acetate extract had the lowest content (0.29 ± 0.191 mg QE/g).

### 3.2. Antioxidant Capacity of Solvent Extracts

The antioxidant activity of the solvent extracts was examined by the ABTS scavenging activity, DPPH scavenging activity, and reducing power assays (Table 2). Ascorbic acid (or vitamin C) is a well-known antioxidant and was used as the positive control in the antioxidant assays. All solvent extracts had an EC_50_ below 1 mg/mL for the scavenging activity on ABTS and DPPH radicals, in which ethanol extract was the lowest for ABTS (0.097 ± 0.00035 mg/mL) and second lowest for DPPH (0.107 ± 0.00598 mg/mL) after ethyl acetate extract (0.052 ± 0.00350 mg/mL). Similarly, all solvent extracts had an EC_50_ below 2 mg/mL for reducing power, with only the ethanol extract showing an EC_50_ below 1 mg/mL. The EC_50_ of ethanol extract in ABTS scavenging activity and reducing power was two-fold lower than ethyl acetate extract. In contrast, EC_50_ of ethanol extract in DPPH scavenging activity was double that of ethyl acetate extract. Overall, ethanol extract had the highest antioxidant activity, followed by ethyl acetate, aqueous, and hexane extract.

### 3.3. Effect of Solvent Extracts on Cell Viability

**Figure 2:**
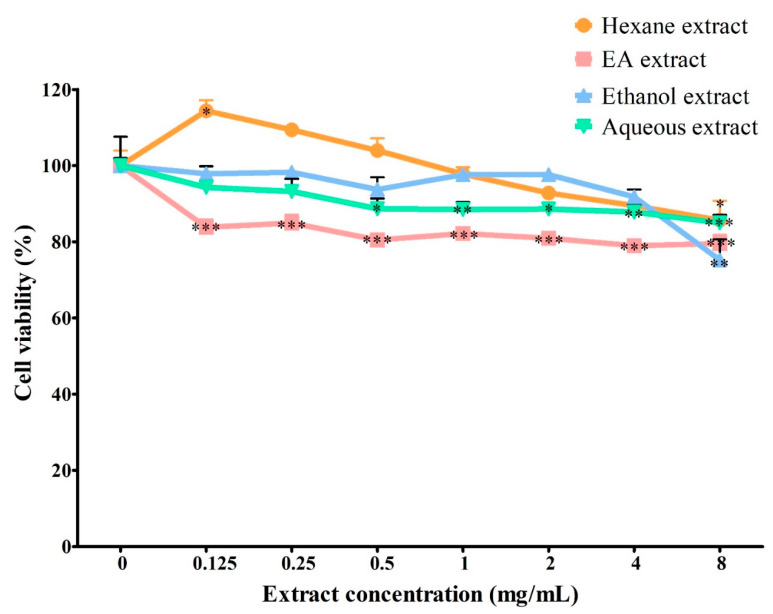
Effect of *S. platensis* extracts on cell viability in BV2 microglia. The cell viability was expressed as a percentage of the negative control (0 mg/mL). Data represent the mean ± SE (*n* = 3). *** *p* ≤ 0.001, ** *p* ≤ 0.01 and * *p* ≤ 0.05 significant difference in cell viability relative to the negative control by Dunnett’s multiple comparison test. EA: ethyl acetate.

As shown in Figure 2, the effect of all solvent extracts on cell viability in BV2 microglia was maintained above 69%, with the cell treated with ethanol extract (8 mg/mL) having the lowest cell viability (75.22 ± 5.40%). However, ethanol extract up to 4 mg/mL maintained cell viability above 90%. Meanwhile, ethyl acetate extract up to 2 mg/mL (80.94 ± 0.43%) and hexane and aqueous extracts at all tested concentrations maintained cell viability above 80%. Extract concentrations with >80% cell viability were chosen for the subsequent study on the anti-neuroinflammatory activity in BV2 microglia.

### 3.4. Solvent Extracts Inhibited LPS-Induced Production of NO

**Figure 3:**
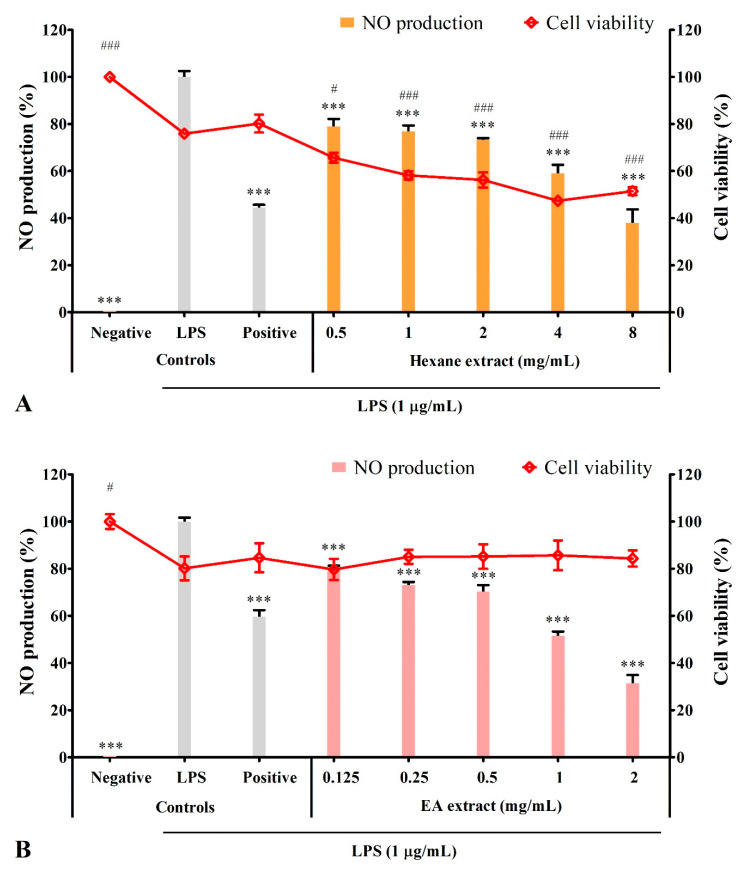

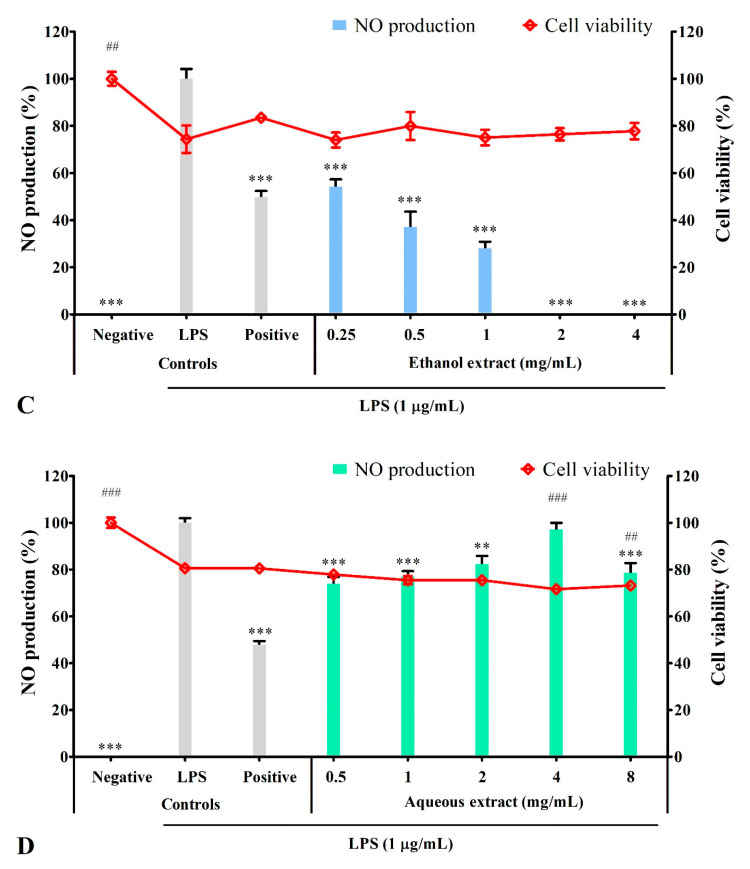

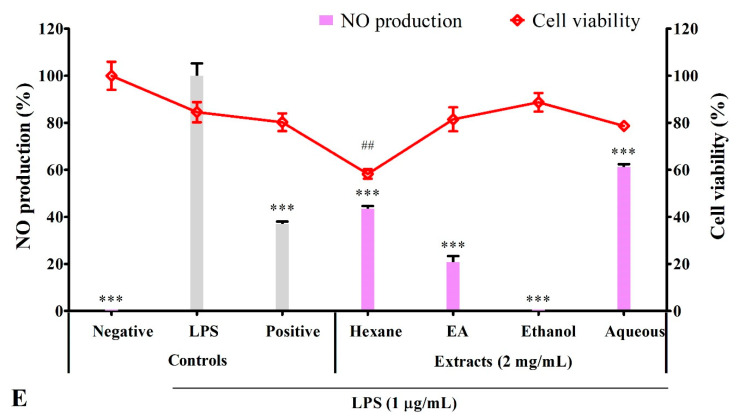
NO inhibitory activity of *S. platensis* solvent extracts in LPS-induced BV2 microglia. Dose-dependent effect of (**A**) hexane, (**B**) ethyl acetate, (**C**) ethanol, (**D**) aqueous extracts, and (**E**) the effect of all solvent extracts at 2 mg/mL on the NO production (bar chart) and cell viability (line chart). NO production and cell viability were expressed as a percentage of the LPS and negative control, respectively. Data represented the mean ± SE (*n* = 3). *** *p* ≤ 0.001 and ** *p* ≤ 0.01 significant in NO production; ### *p* ≤ 0.001, ## *p* ≤ 0.01 and # *p* ≤ 0.05 significant difference in cell viability relative to the LPS control by Dunnett’s multiple comparison test. L-NAME served as the positive control. EA: ethyl acetate.

Under normal conditions, BV2 microglia did not produce NO (negative control). The addition of 1 µg/mL LPS induced NO production (LPS control) ranging from 10.36 to 25.9 µM, with each expressed as 100% NO production. Whereas the positive control L-NAME is a NO inhibitor that inhibited NO production to the range between 36.95% to 59.77% (Figure 3).

Pre-treatment with ethanol or ethyl acetate extracts significantly (*p* ≤ 0.001) inhibited NO production in a dose-dependent manner without significant effect on cell viability at all tested concentrations (Figure 3B,C). At 2 mg/mL, ethanol extract completely inhibited NO production, whereas ethyl acetate extract inhibited NO production to 31.38 ± 3.53%; both showed higher inhibitory activity than the positive control (49.83 ± 2.52% and 59.77 ± 2.67% NO production, respectively). Aqueous extract at all tested concentrations, except 4 mg/mL, significantly (*p* ≤ 0.01) inhibited NO production to the range between 74.02 ± 2.85% to 82.38 ± 3.40%. However, the inhibitory activity was lower than the positive control (47.77 ± 1.67% NO production; Figure 3D). Hexane extract inhibited NO production in a dose-dependent manner. At 8 mg/mL (37.90 ± 5.81% NO production), the inhibitory activity was comparable with the positive control (44.39 ± 1.25% NO production), but the cell viability was reduced by approximately 28.76% (Figure 3A).

At the concentration of 2 mg/mL, only ethanol (0% NO production) and ethyl acetate (20.83 ± 2.53% NO production) extracts showed higher NO inhibitory activity than the positive control (36.95 ± 1.08% NO production; Figure 2E), which corresponded with the dose-dependent result (Figure 3A–D). Overall, ethanol extract had the highest NO inhibitory activity, followed by ethyl acetate and aqueous extracts. Hexane extract did not possess inhibitory activity, as the decrease in NO level might be due to the reduction in the viable cell. Ethanol extract that showed the highest antioxidant and NO inhibitory activities was chosen for the subsequent assays.

### 3.5. Ethanol Extract Inhibited LPS-Induced Production of PGE_2_, TNF-α, and IL-6

**Figure 4:**
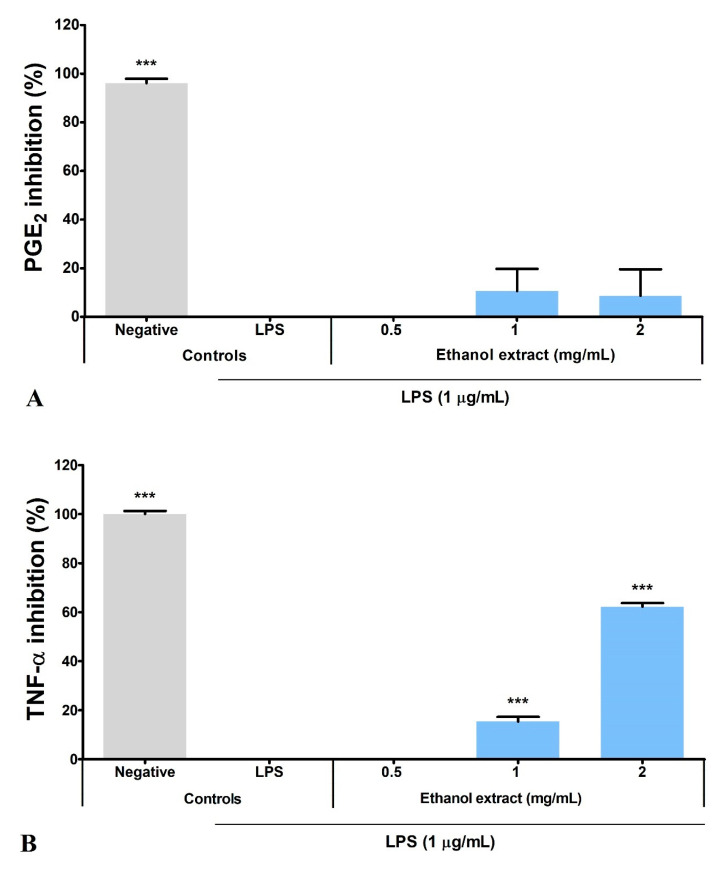

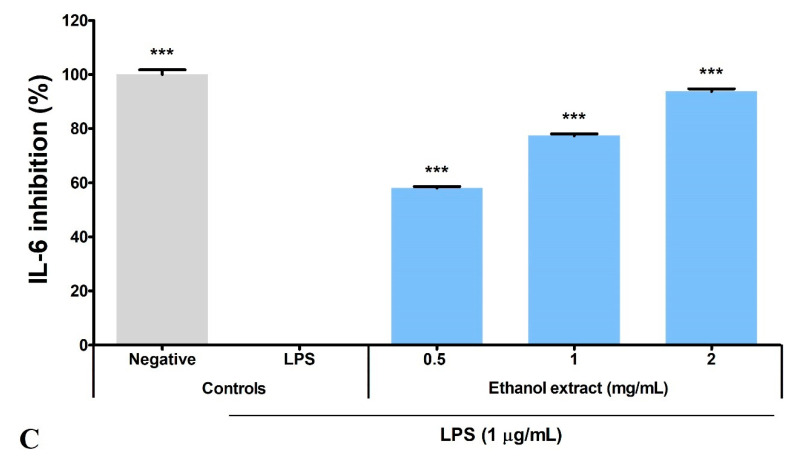
Inhibitory activity of *S. platensis* ethanol extract on the proinflammatory mediator and cytokines production in LPS-induced BV2 microglia. Dose-dependent effects were observed on the production of (**A**) PGE_2_, (**B**) TNF-α and (**C**) IL-6. The inhibitory activities were expressed as a percentage of the negative control. Data represented the mean ± SE (*n* = 3). *** *p* ≤ 0.001 significant difference in inhibitory activity relative to the LPS control by Dunnett’s multiple comparison test.

Overnight LPS stimulation (LPS control) significantly induced a 17.27-fold increase in PGE_2_ production by BV2 microglia and was expressed as zero inhibition percentage. Hence, the basal level of PGE_2_ in BV2 microglia (negative control: 39.21 ± 27.53 pg/mL) was calculated as 96.15% inhibition. Ethanol extract inhibited 10.61 ± 9.15% and 8.64 ± 10.95% PGE_2_ production at 1 and 2 mg/mL, respectively (Figure 4A).

Meanwhile, BV2 microglia did not produce TNF-α and IL-6 (negative controls) under normal conditions. Overnight LPS stimulation (LPS control) induced TNF-α and IL-6 production to 7010 ± 88.32 and 12 912 ± 219.20 pg/mL, respectively. The LPS control was expressed as zero inhibition percentage, while the negative control was 100% inhibition. Ethanol extract significantly (*p* ≤ 0.001) inhibited the TNF-α and IL-6 production in a dose-dependent manner, with higher inhibitory activity on the IL-6 production (Figure 4B,C). Ethanol extract inhibited > 50% of IL-6 production (58.03 ± 0.54% inhibition) at 0.5 mg/mL, but a four-fold increase in concentration was needed to inhibit > 50% of TNF-α production (62.17 ± 1.55% inhibition). Nevertheless, ethanol extract at 2 mg/mL inhibited > 90% of IL-6 production (93.73 ± 1.00% inhibition).

### 3.6. Ethanol Extract Downregulated LPS-Induced Expression of iNOS but Upregulated COX-2

**Figure 5:**
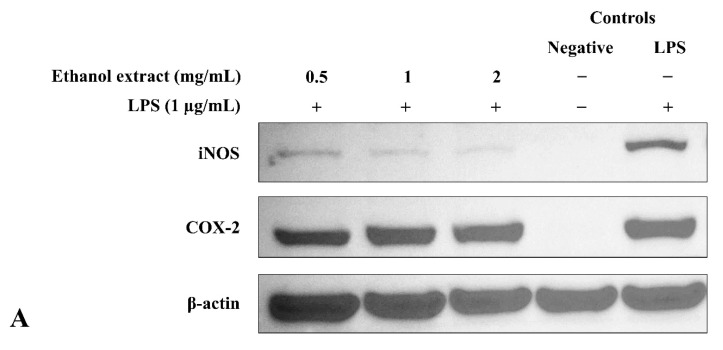

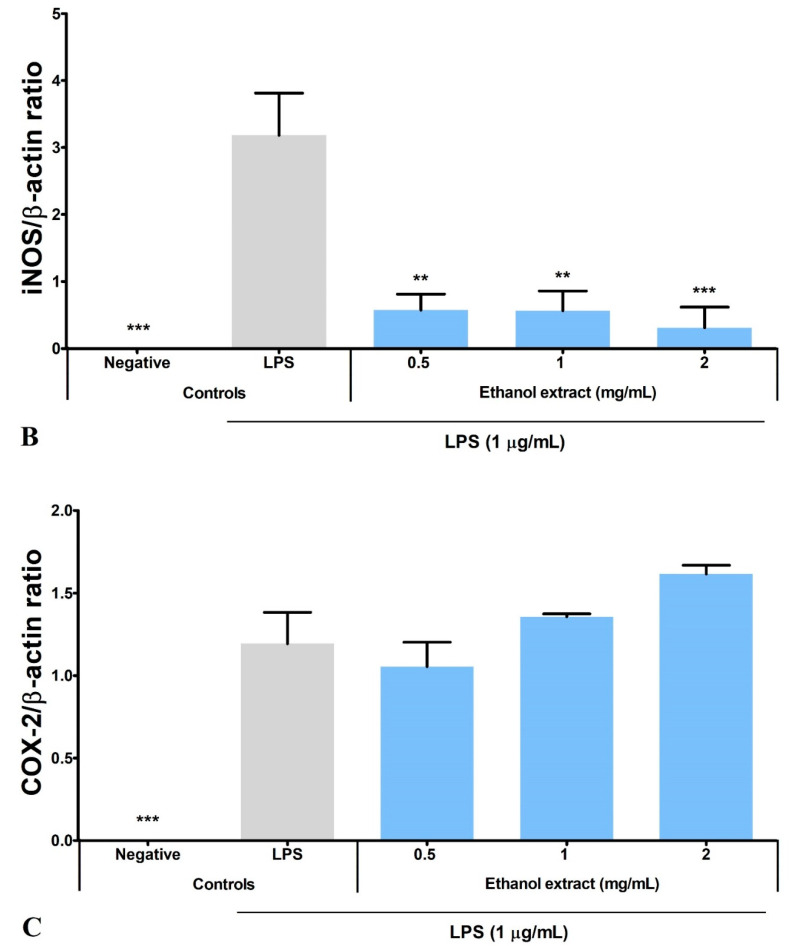
Regulation by *S. platensis* ethanol extract on the expression of the proinflammatory enzymes in LPS-induced BV2 microglia. Dose-dependent effects were observed on (**A**) iNOS and COX-2 protein expression. The blot shown was the representative results of three independent experiments. β-actin served as an internal control. (**B**,**C**) Quantification of relative band intensities from three independent experimental results was determined by densitometry. *** *p* ≤ 0.001 and ** *p* ≤ 0.01 significant difference in protein expression relative to LPS control by Dunnett’s multiple comparison test.

As shown in Figure 5, unstimulated BV2 microglia did not express iNOS and COX-2 (negative controls), whereas overnight LPS stimulation (LPS control) induced the expression of both proteins. Ethanol extract exhibited an opposite effect on the iNOS and COX-2 protein expression in a dose-dependent manner: significantly (*p* ≤ 0.01) inhibited iNOS expression but enhanced COX-2 expression (Figure 5B,C). In relation to the previous results (Figure 3C,E), the NO inhibitory activity of ethanol extract was achieved by downregulating iNOS protein expression.

### 3.7. Bioactive Compounds Profile of Ethanol Extract

The chromatogram showed 59 peaks in ethanol extract (Figure S2), but only 35 compounds had the MFG scores above 90% and ± 5 ppm difference. Twenty-one compounds were tentatively identified in the Metlin database: compound methenamine (i), (morpholinoimino)acetonitrile (ii), benazeprilat (iii), rauwolscine (iv), uncarine C (v), 2-carboxy-4-dodecanolide (vi), 4,5-di-o-methyl-8-prenylafzelechin-4beta-ol (vii), (±)13-azaprostanoic acid (viii), estra-1,3,5(10)-triene-2,17beta-diol (ix), 15(S)-15-methyl PGF2α ethyl amide (x), emmotin A (xi), 3-butylidene-7-hydroxyphthalide (xii), N-cis-tetradec-9Z-enoyl-L-homoserine lactone (xiii), stigmatellin Y (xiv), palmitic amide (xv), 1-monopalmitin (xvi), harderoporphyrin (xvii), hexadecyl acetyl glycerol (xviii), 3α,12α-dihydroxy-5β-chol-8(14)-en-24-oic acid (xix), docosanedioic acid (xx), and hexacosanedioic acid (xxi; Table 3). Emmotin A (xi), palmitic amide (xv), and 1-monopalmitin (xvi) have been reported in studies related to neurodegenerative diseases (Table 3). In fact, emmotin A (xi) and 1-monopalmitin (xvi) are the major compounds in the ethanol extract, represented by the top 2 peaks at 16.743 and 19.299 min, respectively (Figures S2 and S3). The 14 unidentified compounds recorded in Table 4 indicated the presence of unexplored bioactive compounds in *S. platensis*.

## 4. Discussion

*S. platensis* is well-known for its high capacity in antioxidant production. *S. platensis* supplement and polysaccharides protect dopaminergic neurons in the rat models of PD and AD through the regulation of antioxidant and inflammatory mechanisms [26,27]. *S. platensis* supplement and ethanol extract also promote the regeneration of neurons [28,29]. However, this study is the first to study the regulation of neuroinflammation in microglia by the metabolites in the ethanol extract of *S. platensis*. The majority of phytochemicals are secondary metabolites synthesized by plants as part of their defense mechanism [77]. Phytochemicals can be classified into six main groups: carbohydrates, lipids, terpenoids, phenolic, alkaloids, and other nitrogen-containing metabolites [78]. Phenolic compounds have high nutraceutical and pharmaceutical value due to their ability to scavenge ROS, as oxidative stress plays a key role in the progression of multiple diseases, including neurodegenerative diseases [79]. Flavonoids are a subgroup of phenolic compounds, and regular consumption of a flavonoid-rich diet has been reported to reduce the risk of neurodegenerative diseases [80]. The extraction efficiency of the phytochemicals can be influenced by multiple factors such as temperature and incubation period, but solvent for extraction has been the main factor due to the intermolecular forces [81]. In this study, four solvents with different polarities were used for extraction, aimed to maximize the phytochemical extraction from *S. platensis*. The amount of TPC and TFC extracted by solvents with high polarity (water and ethanol) were significantly higher than solvent with lower polarity (ethyl acetate and hexane), as expected since polyphenols are hydrophilic [82]. A study found that phenolic compounds, mainly flavonoids, contributed to the 2.5% dry weight of the polar extract of *S. platensis* [83]. Based on the high-performance liquid chromatography (HPLC) studies, phenolic compounds in *S. platensis* can be categorized into four subgroups: (a) polyphenol (phloroglucinol, resveratrol and pyrogallol); (b) phenolic acids (protocatechuic, succinic, quinic, 4-hydroxybenzoic, citric, vanillic, salicylic, syringic, gallic, caffeic, chlorogenic, rosmarinic, *p*-coumaric, ferulic, and hydroxycinnamic acid); (c) aldehyde (4-hydroxybenzaldehyde and 3,4-dihydroxybenzaldehyde); and (d) flavonoids (apigenin, catechin, rutin, quercetin and quercitrin) [84,85,86].

Based on our results, ethanol extract with the highest TFC also showed the highest ABTS scavenging activity and reducing power. In comparison, DPPH scavenging activity was the highest in ethyl acetate extract, followed by ethanol, hexane, and aqueous extracts. These results inferred that the flavonoids in *S. platensis* contribute to the ABTS scavenging activity and reducing power but not DPPH scavenging activity since hexane extract with zero flavonoid content showed higher activity than aqueous extract. This was justified when ethyl acetate extract with low TFC (0.29 ± 0.191 mg QE/g) showed the highest DPPH scavenging activity. In agreement with Bellahcen et al., we found that organic solvents showed higher efficacy in the extraction of antioxidants from *S. platensis* compared to water [87]. Furthermore, organic solvents with high polarity have a higher capability in the extraction of antioxidants compared to non-polar organic solvents. This is consistent with the reported finding, which showed *S. platensis* ethanol extract exhibited higher antioxidant activity than hexane extract [88]. This finding also applies to macroalgae, where a similar finding has been reported in the brown macroalga *Padina australis* [89]. A study that utilized both thin-layer chromatography (TLC) and HPLC-diode array detection (DAD) reported that active antioxidants in *S. platensis* ethanol extract include β-carotene, zeaxanthin, carotenoids, and phenolic compounds [88]. LC-MS analysis also identified a list of potential antioxidants in *S. platensis*, including carotenoids (siphonein, zeaxanthin, myxoxanthophyll fucoside, astaxanthin, and β-carotene), chlorophyll and the derivatives (chlorophyll a, pheophytin a, pyropheophytin a, and pheophorbide a) and phenolic compounds [90]. Both carotenoids and phenolic compounds are popular antioxidants [77,91]. As such, carotenoids may be the active compound for DPPH scavenging activity in the solvent extracts since flavonoid has been excluded (as mentioned above). Regardless of the DPPH scavenging activity, our results suggested flavonoids as the predominant antioxidant in *S. platensis*. It had been presumed that microalgae were unable to synthesize flavonoids until the genes involved in the flavonoid synthesis pathway were detected in microalgae in 2008 [84]. Hence, further investigations are essential to gather sufficient information on the flavonoid synthesis in *S. platensis*.

**Figure 6:**
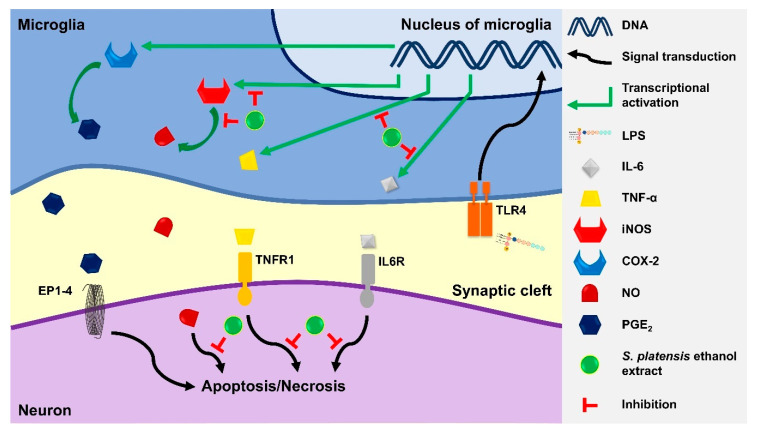
Illustration of neuroprotective effect by *S. platensis* ethanol extract via downregulation of the LPS-induced production of proinflammatory mediators and cytokines in BV2 microglia. TLR4: Toll-like receptor 4; EP1-4: Prostaglandin E2 receptor 1-4; TNFR1: Tumor necrosis factor receptor 1; IL6R: Interleukin 6 receptor.

The neuroinflammatory response is a cascade of proinflammatory mediators and cytokine production by neuroglia until the resolution mechanism takes place. Proinflammatory cytokines or ROS trigger the expression of enzymes, iNOS, and COX-2, in microglia to produce NO and PGE_2,_ respectively [92,93]. Overproduction of NO can be detrimental to neuronal cells by increasing the level of ROS to prolong the neuroinflammatory cascade [94], reacting with superoxide radicals to produce neurotoxins, and modifying the proteins to promote neuronal cell death and protein aggregation [95,96]. Similarly, PGE_2_ binds to the receptors located on microglia to amplify the production of proinflammatory cytokines [93]. Overproduction of proinflammatory cytokines, such as TNF-α, can activate the caspase cascade, which results in neuronal apoptosis [97]. As illustrated in Figure 6, we found that ethanol extract inhibited neuroinflammation by reducing the NO production via downregulation of iNOS protein expression in LPS-induced BV2 microglia. Ethanol extract also reduced the production of TNF-α and IL-6 in a dose-dependent manner but showed no significant inhibition on neither the PGE_2_ production nor COX-2 protein expression. Studies have reported that the acetone and ethyl acetate extracts of *S. platensis* also reduced the expression of iNOS, TNF-α and IL-6, with the ethyl acetate extract suppressing the expression of COX-2 [98,99]. COX-2 has been labeled as a proinflammatory mediator since it was mainly expressed during inflammation, but its role in neuroinflammation remains controversial.

Studies reported that the absence of COX-2 favored the proinflammatory response [100], and acetylation of COX-2 induced the production of anti-inflammatory mediators [101]. Chen et al. (2012) reported that the expression of iNOS, COX-2, TNF-α, and IL-6 was inhibited by C-phycocyanin (C-PC) present in *S. platensis* [102]. Since our ethanol extract did not inhibit the expression of COX-2, we can safely assume that C-PC is not the responsible bioactive compound in the ethanol extract. Inhibitory activity on the production of NO, PGE_2_, TNF-α, IL-1β and IL-6 by ethanol extract of another species, *S. maxima,* increased as the concentration of chlorophylls increased, indicating the potential of chlorophylls in *Spirulina* as anti-neuroinflammatory agent [103]. Carotenoids, another group of natural pigments isolated from the microalgae *Nitzschia laevis* and *Euglena gracilis*, also exhibited similar anti-neuroinflammatory activities [104,105].

The tentatively identified compounds in ethanol extract comprise different groups of metabolites: amines, nitriles, dipeptides, alkaloids, γ-butyrolactones, phenolic compounds, fatty acids, terpenoids, phthalides, porphyrins, glycerolipids, and ethers. We proposed emmotin A (terpenoid), palmitic amide (fatty acid amide) and 1-monopalmitin (glycerolipid) that involved in neurodegenerative diseases as the bioactive compounds in ethanol extract. Terpenoids are a major group of phytochemicals, with carotenoids and steroids as the subgroups [106]. Emmotin A showed strong binding interaction with acetylcholinesterase (AChE) and butyrylcholinesterase (BChE) in an in silico molecular docking study [51]. Other terpenoids with positive interaction in the study showed in vitro AChE inhibitory and antioxidant activities [107]. As mentioned above, the FDA-approved drugs for AD ameliorated cognitive impairment by inhibiting the breakdown of ACh by both AChE and BuChE [11]. Furthermore, these drugs suppressed microglia activation and inhibited proinflammatory cytokines production, supporting the role of cholinergic neurotransmission in the regulation of neuroinflammation [108]. These studies suggested the neuroprotective potential of emmotin A by maintaining the level of ACh and suppressing neuroinflammation. Similarly, fucosterol, a steroid isolated from *P. australis,* has been reported with anti-cholinesterase and anti-neuroinflammatory activity in BV2 microglia [109]. Astaxanthin [110], fucoxanthin [111], and fucoxanthinol [112] are marine carotenoids reported to inhibit the production of NO and proinflammatory cytokines through the regulation of nuclear factor kappa B (NF-κB) pathway. The anti-neuroinflammatory and antioxidant activities of fucoxanthin and fucoxanthinol also involved the regulation of mitogen-activated protein kinase (MAPK) and nuclear erythroid 2-related factor 2 (Nrf2)/heme oxygenase-1 (HO-1) pathways [104,111].

Lipids play an important role in brain development and can be divided into eight subgroups: fatty acids, glycerolipids, glycerophospholipids, sphingolipids, sterol lipids, prenol lipids, saccharolipids, and polyketides [112]. Palmitic amide is a ligand of peroxisome proliferator-activated receptor alpha (PPARα) with the capability to penetrate CNS and promote the synaptic function of hippocampal neurons via upregulation of the cAMP-response element-binding protein (CREB) [59,113]. PPARα is a transcription factor with neuroprotective effects reported in various neurological disease models, including AD and PD. The effect was mainly attributed to the antioxidant and anti-neuroinflammatory activities [108]. Palmitic amide and 1-monopalmitin have been identified as a biomarker for AD [57] and CNS inflammatory demyelinating diseases (IDDs) [61], respectively. CNS IDDs are a group of diseases with inflammatory lesions, including multiple sclerosis. 1-Monopalmitin was upregulated in the cerebrospinal fluid of CNS IDDs patients, indicating that 1-monopalmitin plays a role in neuroinflammation [61]. Recently, 1-monopalmitin has been identified in the methanol extract of a red macroalga *Kappaphycus malesianus*, that suppressed the proinflammatory mediators and cytokines production by downregulating the protein kinase B (AKT)/NF-κB and extracellular signal-regulated kinase 1 and 2 (ERK1/2) pathways [114]. Despite the lack of study on palmitic amide and 1-monopalmitin, other algae-derived lipids have shown neuroprotective effects by modulating the proinflammatory mediators and cytokines. Hexadecanoic acid, a fatty acid isolated from *Myagropsis myagroides,* inhibited the production of proinflammatory mediators and cytokines via NF-κB, ERK1/2, and c-Jun NH2-terminal kinase (JNK) pathways in BV2 microglia [115]. Besides macroalgae, lipids isolated from *Chlorella sorokiniana* and *Tetraselmis chui* also showed NO inhibitory activity in BV2 microglia [116,117]. Lipid extracts from *Porphyra dioica, Palmaria palmata, Chondrus crispus,* and *Pavlova lutheri* downregulated the expression of 14 proinflammatory genes in LPS-induced human THP-1 macrophages and inhibited the production of IL-8. However, only extracts from *P. lutheri* and *P. palmata* showed inhibition on IL-6 and TNF-α [118].

## 5. Conclusions

Our result revealed that *S. platensis* ethanol extract possesses anti-neuroinflammatory activity by regulating the production of proinflammatory mediators and cytokines in LPS-induced BV2 microglia. Emmotin A, palmitic amide, and 1-monopalmitin were proposed as potential bioactive compounds in our ethanol extract with bioactivity linked to neuroinflammation. However, further investigation on bioassay-guided isolation and characterization is required for the identification of the bioactive compound(s). In addition, investigation of the underlying mechanisms and in vivo study involving the proposed bioactive compounds are essential for the development of the compounds into functional food. Currently, the field of nutrition is working towards precision nutrition to provide tailored nutritional advice in the prevention and management of the disease. This study serves as the preliminary assessment for the compound(s) isolation and characterization, which is essential in the development of precision nutrition.

## Figures and Tables

**Table 1:** Yield percentage and phytochemical content of *S. platensis* extracts.

Extract	Yield (%)	TPC (mg GAE/g)	TFC (mg QE/g)
Hexane	2.98 ± 0.442 ^a^	4.63 ± 0.594 ^a^	0.00 ± 0.000 ^a^
Ethyl acetate	5.31 ± 0.766 ^a^	10.26 ± 0.864 ^b^	0.29 ± 0.191 ^a^
Ethanol	3.45 ± 0.042 ^a^	27.35 ± 0.400 ^c^	83.41 ± 2.049 ^b^
Aqueous	27.75 ± 0.877 ^b^	32.40 ± 0.515 ^d^	7.24 ± 0.389 ^c^

Data expressed as mean ± standard error (SE; *n* = 3). Means with different letters indicate significant differences (*p* ≤ 0.05; Tukey’s test).

**Table 2:** Antioxidant activities of *S. platensis* extracts.

Extract/Control	EC_50_ (mg/mL)		
	ABTS	DPPH	Reducing Power
Extract			
Hexane	0.692 ± 0.02157 ^a^	0.175 ± 0.00202 ^a^	1.683 ± 0.02121 ^a^
Ethyl acetate	0.206 ± 0.00060 ^b^	0.052 ± 0.00350 ^b^	1.533 ± 0.01966 ^b^
Ethanol	0.097 ± 0.00035 ^c^	0.107 ± 0.00598 ^c^	0.8078 ± 0.03707 ^c^
Aqueous	0.305 ± 0.01583 ^d^	0.367 ± 0.01493 ^d^	1.025 ± 0.01155 ^d^
Positive control			
Ascorbic acid	0.009 ± 0.00013 ^e^	0.001 ± 0.00002 ^e^	0.008 ± 0.00047 ^e^

Data expressed as mean ± SE (*n* = 3). Means with different alphabets indicate significant differences (*p* ≤ 0.05; Tukey’s test). A lower EC_50_ (half-maximum effective concentration) indicates a higher antioxidant activity. Ascorbic acid served as the positive control.

**Table 3:** Tentatively identified compounds in *S. platensis* ethanol extract.

No.	Compound Name	Molecular Formula	Molecular Mass	*m*/*z* Ratio [Ion]	Molecular Structure	Classification	Bioactivity
i	Methenamine	C_6_H_12_N_4_	140.106	141.1132 [M + H]^+^	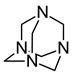	Amines	FDA-approved antiseptic agent to prevent recurrent urinary tract infections [33]. Anticancer activity in systemic cancers and glioblastoma [34].
ii	(Morpholinoimino)acetonitrile	C_6_H_9_N_3_O	139.0742	157.1081 [M + NH_4_]^+^	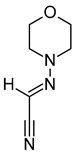	Nitriles	Protect against ischemia-reperfusion injury [35].
iii	Benazeprilat	C_22_H_24_N_2_O_5_	396.1687	397.1761 [M + H]^+^	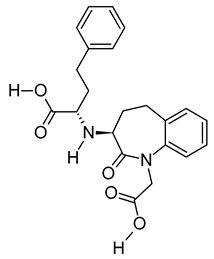	Dipeptide	An ACEi that protects against diabetic cardiomyopathy [36] and hypertension [37].
iv	Rauwolscine	C_21_H_26_N_2_O_3_	354.1938	355.2013 [M + H]^+^	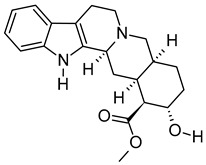	Alkaloid	An α_2C_-adrenoceptor antagonist that protects against peripheral antinociception [38], hypertension [39], myocardial ischemia [40], hyperactivity [41], psychosis [42] and breast cancer [43].
v	Uncarine C	C_21_H_24_N_2_O_4_	368.1734	369.1806 [M + H]^+^	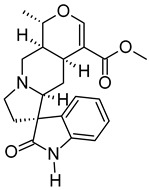	Alkaloid	Antigenotoxic, antioxidant, and immunomodulatory activity [44]. Anticancer activity in medullary thyroid cancer [45], bladder cancer [46], and lymphoblastic leukemia [47].
vi	2-Carboxy-4-dodecanolide	C_13_H_22_O_4_	242.152	243.1593 [M + H]^+^	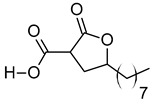	γ-butyrolactone	N/A
vii	4,5-Di-O-methyl-8-prenylafzelechin-4beta-ol	C_22_H_26_O_6_	386.1734	404.2063 [M + NH_4_]^+^	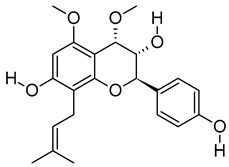	Flavonoid	N/A
viii	(±)13-Azaprostanoic acid	C_19_H_37_NO_2_	311.2822	329.3163 [M + NH_4_]^+^	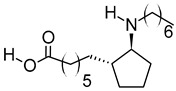	Fatty acid	Antagonist of thromboxane/endoperoxide receptor that protects against thrombosis [48], hypertension [49], and endotoxic shock [50].
ix	Estra-1,3,5(10)-triene-2,17beta-diol	C_18_H_24_O_2_	272.1774	273.1848 [M + H]^+^	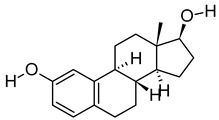	Terpenoid	N/A
x	15(S)-15-methyl PGF2α ethyl amide	C_23_H_41_NO_4_	395.3029	396.3106 [M + H]^+^	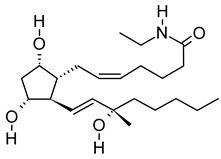	N/A	N/A
xi	Emmotin A	C_16_H_22_O_4_	278.1521	279.1594 [M + H]^+^	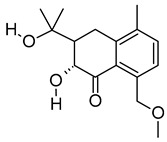	Terpenoid	An enzyme inhibitor that has binding interaction with AChE, BChE, α-glucosidase, α-amylase, and tyrosine [51].
xii	3-Butylidene-7-hydroxyphthalide	C_12_H_12_O_3_	204.0786	205.0858 [M + H]^+^	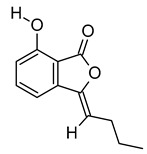	Phthalide	Anticancer activity in human small cell lung cancer [52].
xiii	N-cis-tetradec-9Z-enoyl-L-Homoserine lactone	C_18_H_31_NO_3_	309.2303	310.2374 [M + H]^+^	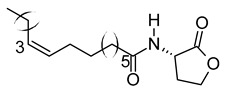	Lactone	N/A
xiv	Stigmatellin Y	C_29_H_40_O_6_	484.2826	502.3166 [M + NH_4_]^+^	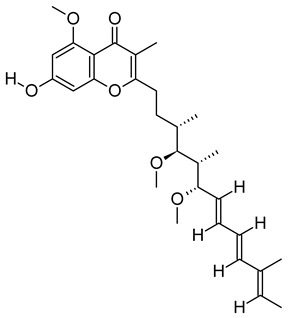	Phenolic	Inhibit the virulence of *Pseudomonas aeruginosa* [53].
xv	Palmitic amide	C_16_H_33_NO	255.2562	256.2636 [M + H]^+^	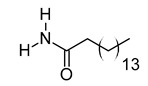	Fatty acid amide	Biomarker for diabetes [54], PCOS [55], psoriasis vulgaris [56], AD [57] and alcoholism [58]. A ligand of PPARα that upregulates synaptic function in hippocampal neurons [59].
xvi	1-monopalmitin	C_19_H_38_O_4_	330.2773	353.2669 [M + Na]^+^	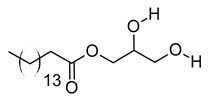	Glycerolipid	Antidiabetic activity [60]. Biomarker for CNS iDDs [61], mycotoxins exposure [62], PCOS with complications [63], and stem cell transplantation recipients [64].
xvii	Harderoporphyrin	C_35_H_36_N_4_O_6_	608.2636	609.2708 [M + H]^+^	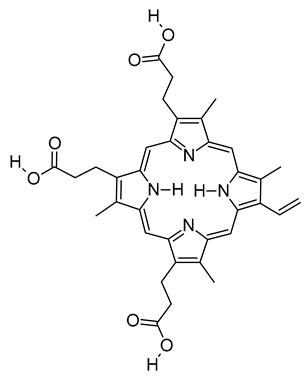	Porphyrin	Biomarker for harderoporphyria [65].
xviii	Hexadecyl acetyl glycerol	C_21_H_42_O_4_	358.3092	381.2983 [M + Na]^+^	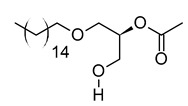	Ether	Induce differentiation in human promyelocytic leukemia cell line HL-60 [66]. Inhibit platelet aggregation [67]. Modulate PKC activity [68].
xix	3α,12α-Dihydroxy-5β-chol-8(14)-en-24-oic Acid	C_24_H_38_O_4_	390.278	391.2854 [M + H]^+^	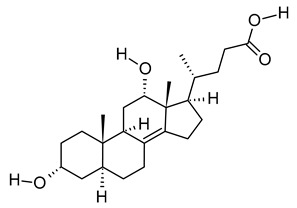	Terpenoid	Biomarker for acetaminophen-related toxicity [69] and colon cancer [70].
xx	Docosanedioic acid	C_22_H_42_O_4_	370.3077	371.3151 [M + H]^+^	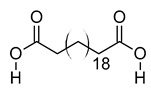	Fatty acid	Antioxidant activity in human HT-29 colon cancer cells [71]. Bactericidal activity against *P. aeruginosa* [72]. Biomarker for Sjogren–Larsson syndrome [73] and patients exposed to TCDD [74].
xxi	Hexacosanedioic acid	C_26_H_50_O_4_	426.3705	449.3598 [M + Na]^+^	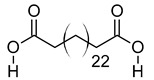	Fatty acid	Biomarker for chronic kidney disorder [75] and poor pregnancy prediction [76].

**Table 4:** Unknown compounds present in *S. platensis* ethanol extract.

No.	Molecular Formula	Molecular Mass	m/z Ratio	Ion
i	C_36_H_66_N_6_O_6_	678.5048	679.5121	[M + H]^+^
ii	C_13_H_20_O_4_	240.1365	241.1435	[M + H]^+^
iii	C_9_H_19_NO	157.1463	158.1536	[M + H]^+^
iv	C_8_H_4_O_3_	148.0157	149.023	[M + H]^+^
v	C_18_H_38_O_4_	318.2766	336.3108	[M + NH_4_]^+^
vi	C_16_H_34_O_3_	274.2506	275.258	[M + H]^+^
vii	C_37_H_74_N_2_O_7_S	690.5213	691.5291	[M + H]^+^
viii	C_18_H_38_O_3_	302.2813	325.2736	[M + Na]^+^
ix	C_35_H_42_O_10_	622.2766	623.2835	[M + H]^+^
x	C_36_H_38_N_4_O_5_	606.2846	607.2916	[M + H]^+^
xi	C_37_H_40_N_4_O_5_	620.2998	621.3072	[M + H]^+^
xii	C_38_H_36_N_8_O	620.3005	621.3076	[M + H]^+^
xiii	C_38_H_51_N_3_O	565.4037	566.4108	[M + H]^+^
xiv	C_44_H_58_N_2_O_3_	662.4455	663.4537	[M + H]^+^

## Data Availability

The data presented in this study are available in article and supplementary material.

## Supplementary Materials

The following supporting information can be downloaded at: https://www.mdpi.com/article/10.3390/metabo12111147/s1, Figure S1: Original Western blot images for three repeats of iNOS, COX-2 and β-actin; Figure S2: Chromatogram of *S. platensis* ethanol extract; Figure S3: Mass spectra of individual compounds.
