# Supplementary material for: Spirulina platensis Suppressed iNOS and Proinflammatory Cytokines in Lipopolysaccharide-Induced BV2 Microglia
Source: Metabolites. 2022 Nov 20;12(11):1147. doi: 10.3390/metabo12111147 (PMC9698046; doi:10.3390/metabo12111147)

Figure S1: Original Western blot images for three repeats of iNOS, COX-2 and β-actin

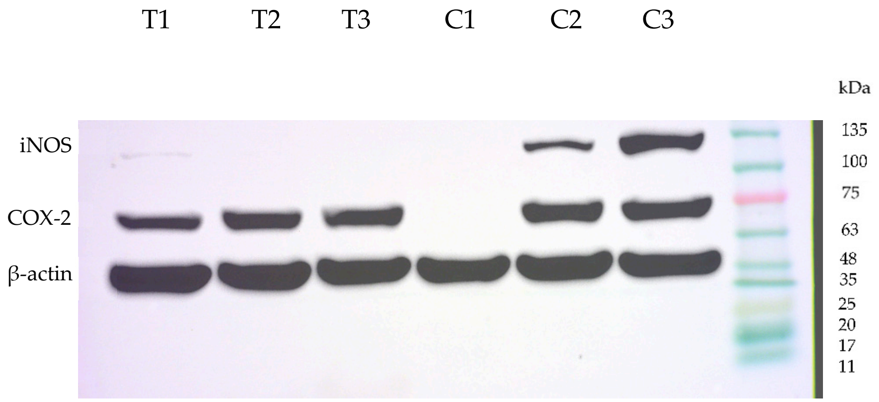

Representative blot shown in main text (Repeat 1)

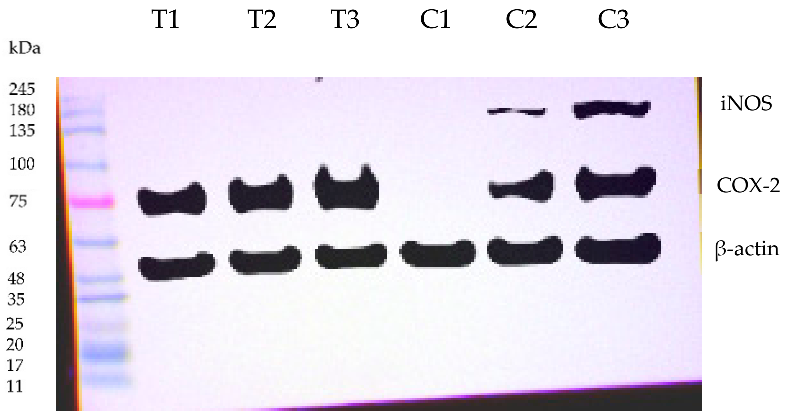

Repeat 2

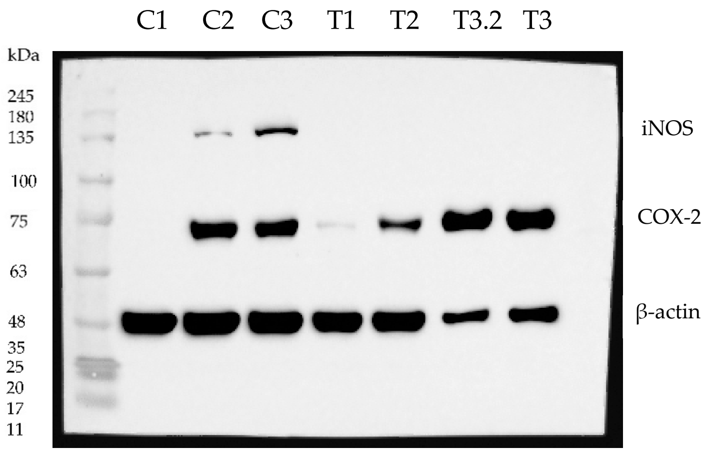

Repeat 3

T1: *Spirulina platensis* ethanol extract 0.5 mg/mL
T2: *Spirulina platensis* ethanol extract 1 mg/mL
T3: *Spirulina platensis* ethanol extract 2 mg/mL
C1: Negative control (Untreated)
C2: Lipopolysaccharide (LPS) control (Stimulated with 1 μg/mL LPS)

Excluded in the main text:
C3: Positive control (Stimulated with 1 μg/mL LPS + treated with N(γ)-nitro-L-arginine methyl ester (L-NAME))
T3.2: Replicate of *Spirulina platensis* ethanol extract 2 mg/mL

Molecular weight of the target proteins:
iNOS – 130 kDa
COX-2 – 74 kDa
β-actin – 45 kDa

Figure S2: Chromatogram of *S. platensis* ethanol extract

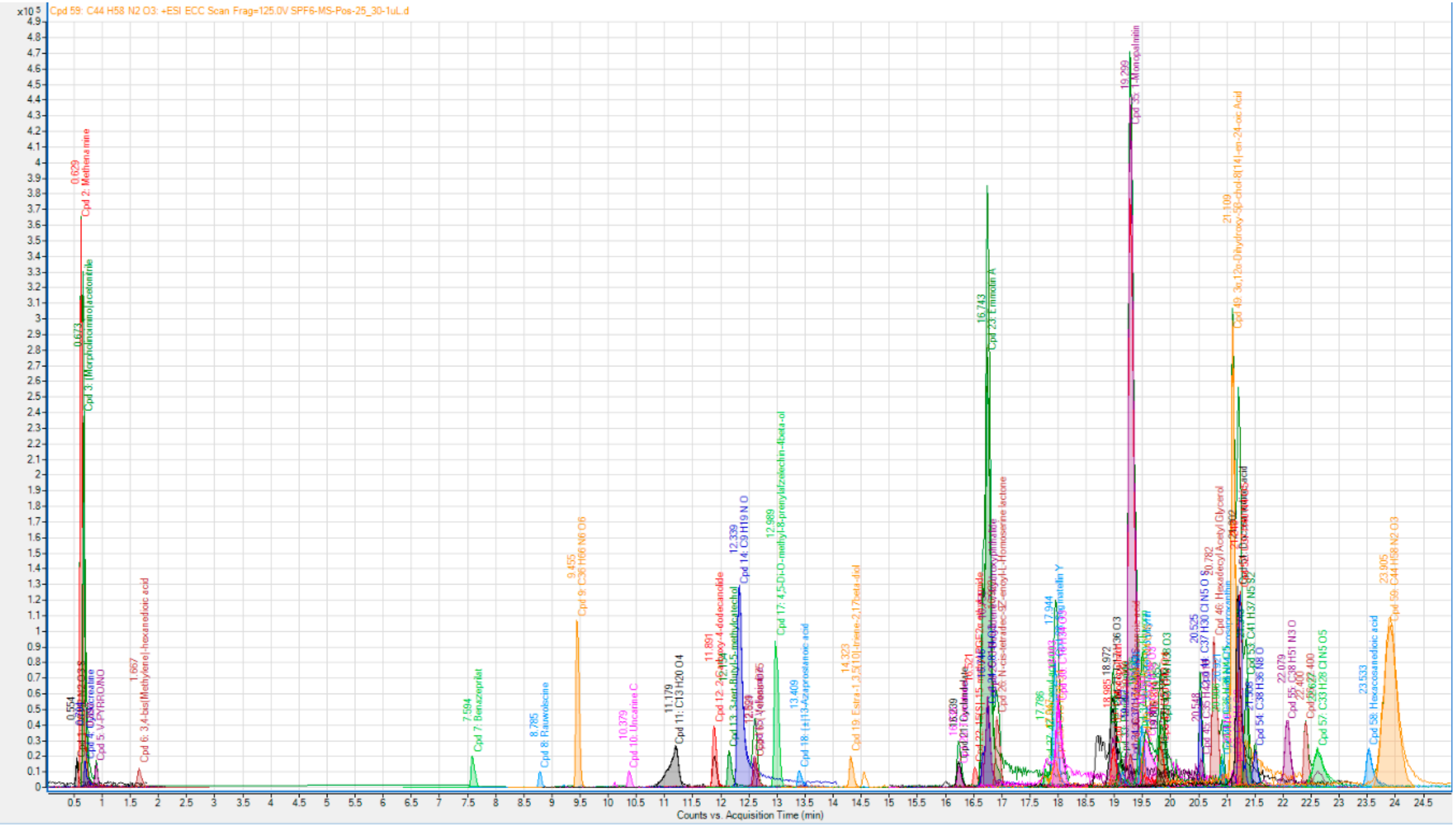

Figure S3: Mass spectra of individual compounds

Compound i: Methenamine

Compound Label	Name	*m/z*	RT	Algorithm	Mass
Cpd 2: Methenamine	**Methenamine**	141.1132	0.629	Find by Molecular Feature	140.106

Compound Chromatograms

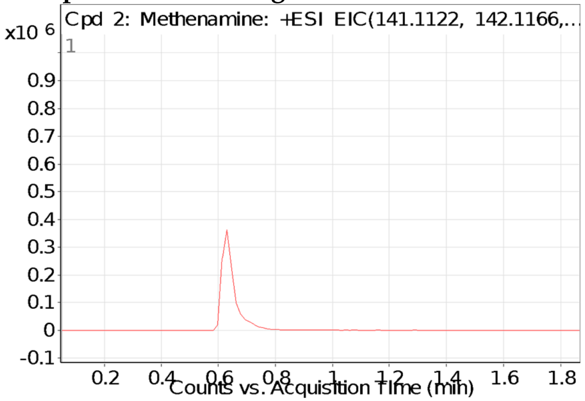

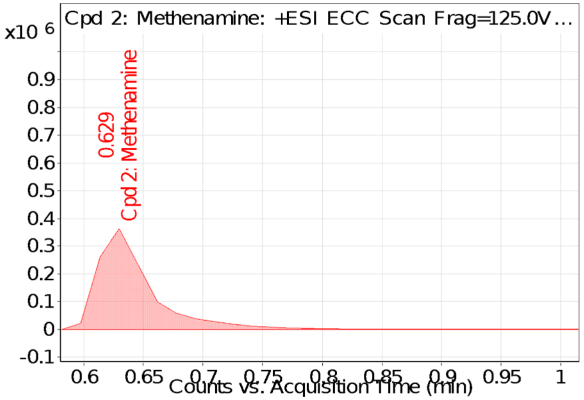

MFE MS Spectrum

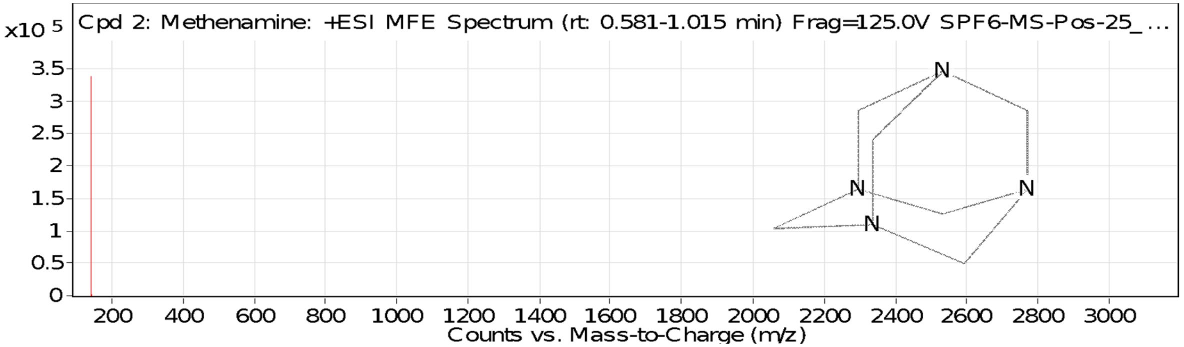

<u>Compound ii: Morpholinoiminoacetonitrile</u>

Compound Label	Name	*m/z*	RT	Algorithm	Mass
Cpd 3: (Morpholinoimino)acetonitrile	**(Morpholinoimino)acetonitrile**	157.1081	0.673	Find by Molecular Feature	139.0742

Compound Chromatograms

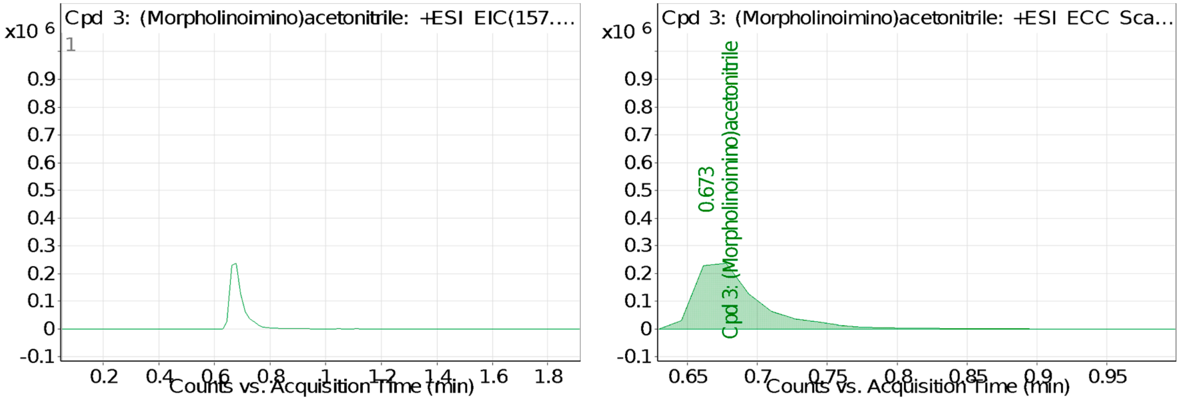

MFE MS Spectrum

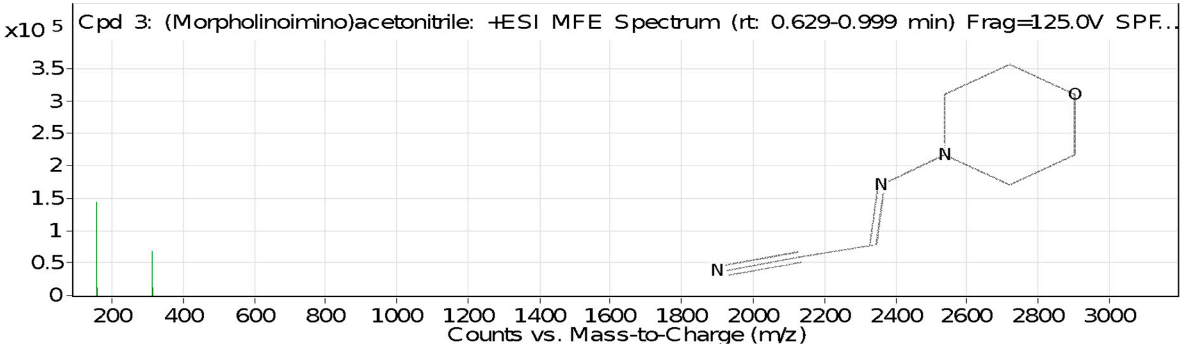

Compound iii: Benazeprilat

Compound Label	Name	*m/z*	RT	Algorithm	Mass
Cpd 7: Benazeprilat	**Benazeprilat**	397.1761	7.594	Find by Molecular Feature	396.1687

Compound Chromatograms

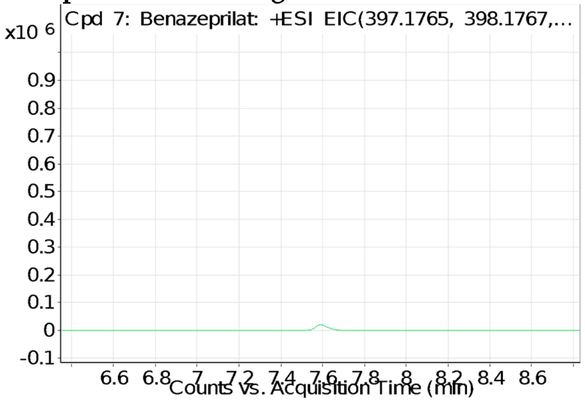

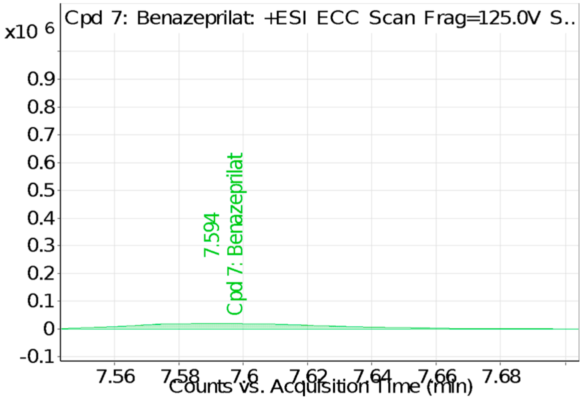

MFE MS Spectrum

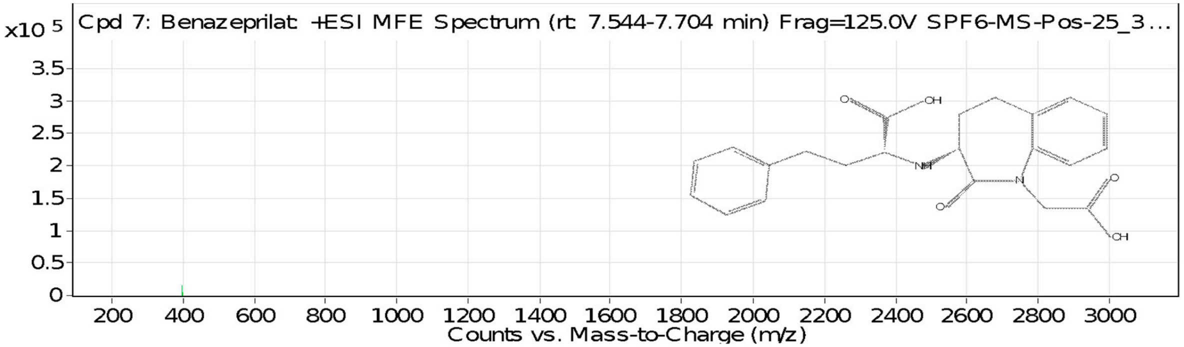

Compound iv: Rauwolscine

Compound Label	Name	*m/z*	RT	Algorithm	Mass
Cpd 8: Rauwolscine	**Rauwolscine**	355.2013	8.785	Find by Molecular Feature	354.1938

Compound Chromatograms

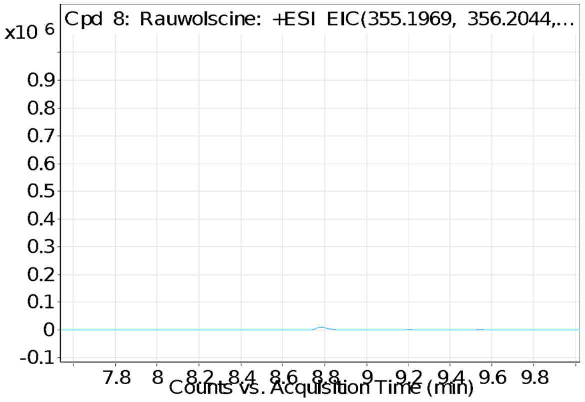

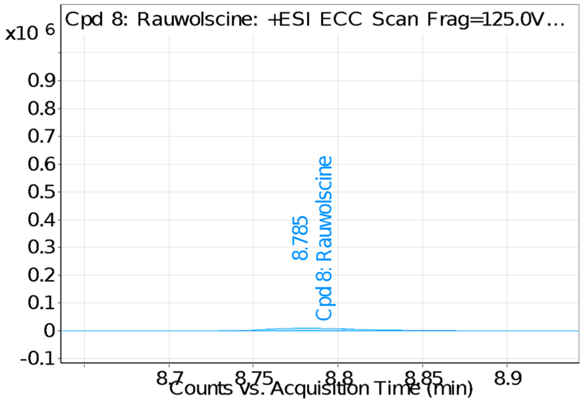

MFE MS Spectrum

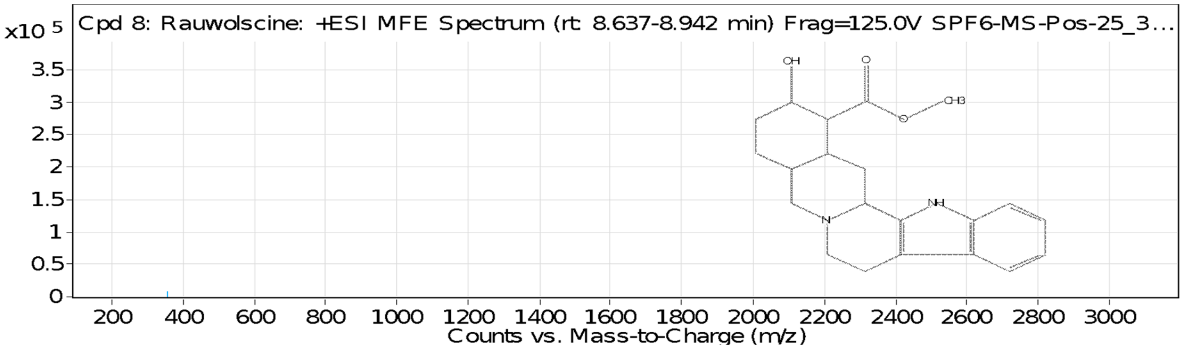

Compound v: Uncarine C

Compound Label	Name	*m/z*	RT	Algorithm	Mass
Cpd 10: Uncarine C	**Uncarine C**	369.1806	10.379	Find by Molecular Feature	368.1734

Compound Chromatograms

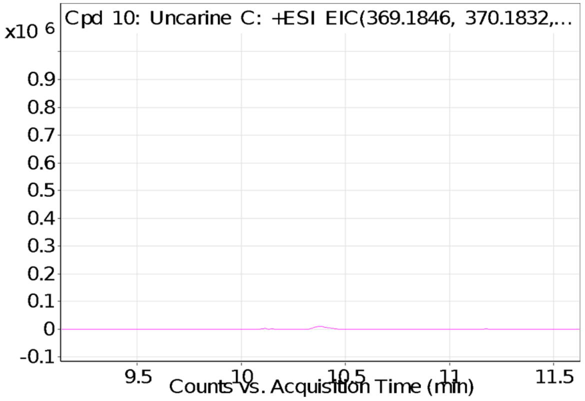

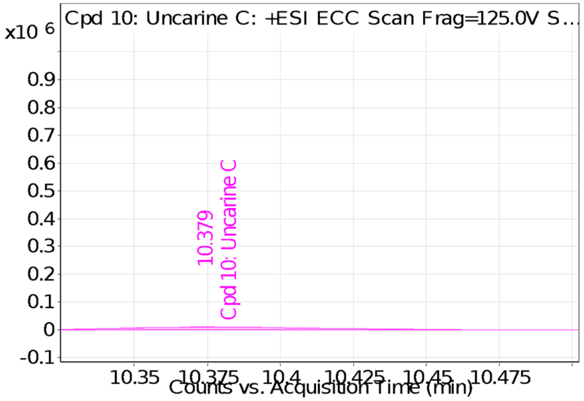

MFE MS Spectrum

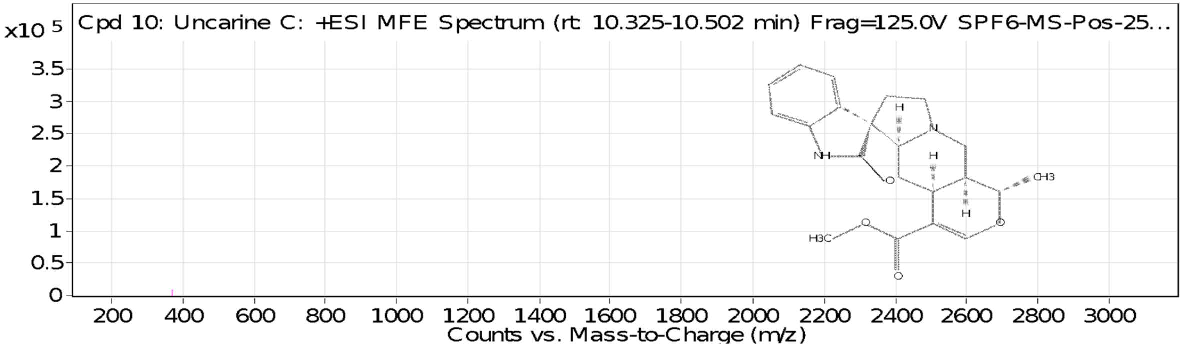

Compound vi: 2-Carboxy-4-dodecanolide

Compound Label	Name	*m/z*	RT	Algorithm	Mass
Cpd 12: 2-Carboxy-4-dodecanolide	**2-Carboxy-4-dodecanolide**	243.1593	11.891	Find by Molecular Feature	242.152

Compound Chromatograms

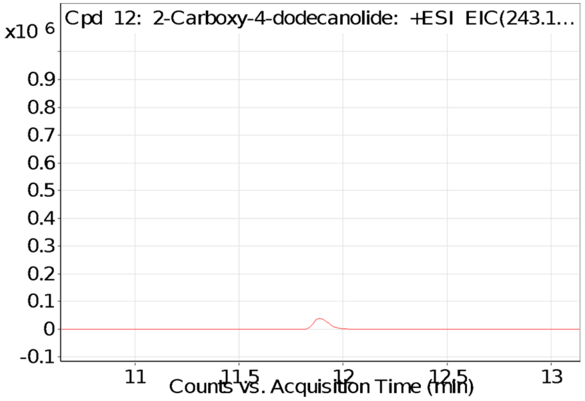

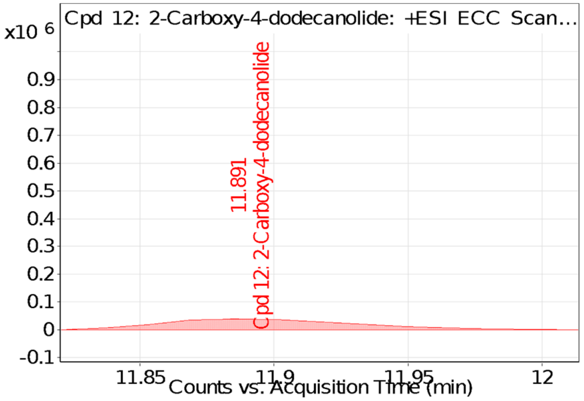

MFE MS Spectrum

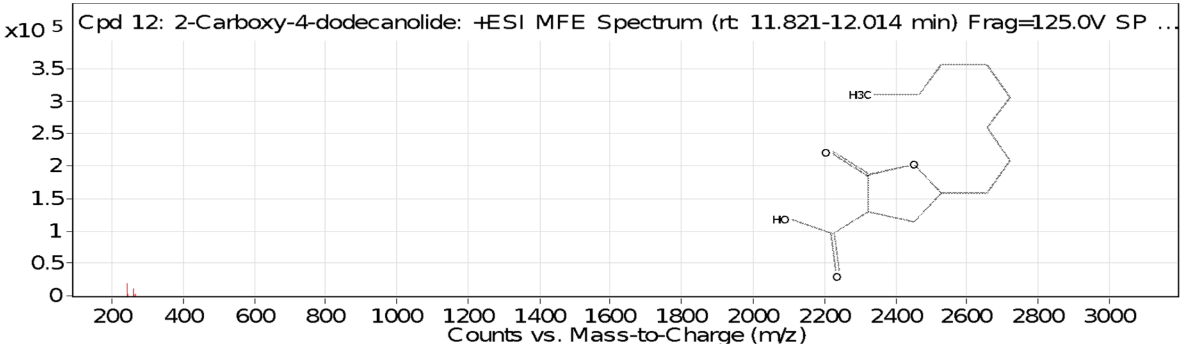

Compound vii: 4,5-Di-O-methyl-8-prenylafzelechin-4beta-ol

Compound Label	Name	*m/z*	RT	Algorithm	Mass
Cpd 17: 4,5-Di-O-methyl-8-prenylafzelechin-4beta-ol	**4,5-Di-O-methyl-8-prenylafzelechin-4beta-ol**	404.2063	12.989	Find by Molecular Feature	386.1734

Compound Chromatograms

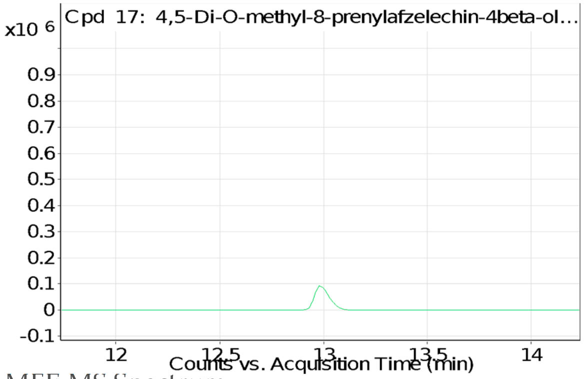

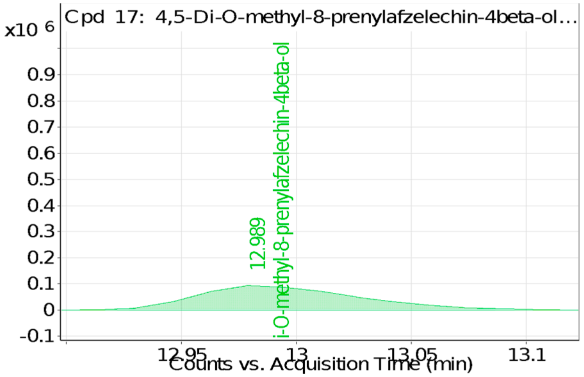

MFE MS Spectrum

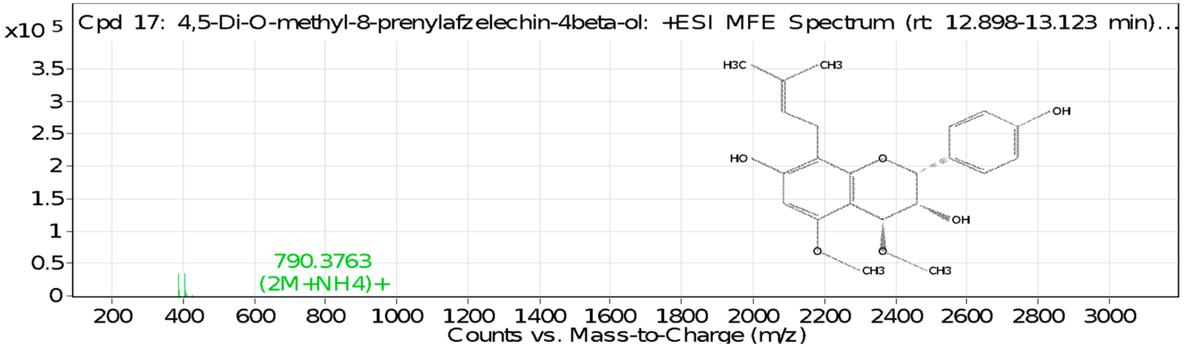

Compound viii: (±)13-Azaprostanoic acid

Compound Label	Name	*m/z*	RT	Algorithm	Mass
Cpd 18: (±)13-Azaprostanoic acid	**(±)13-Azaprostanoic acid**	329.3163	13.409	Find by Molecular Feature	311.2822

Compound Chromatograms

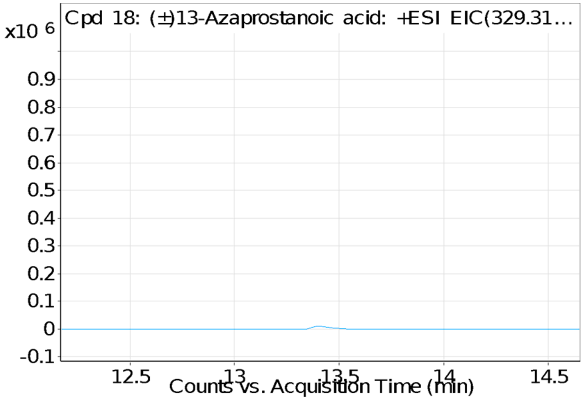

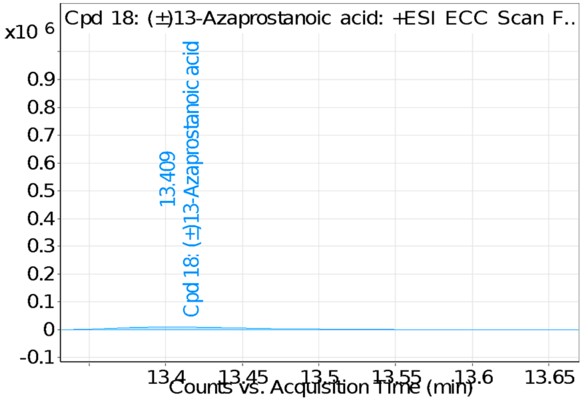

MFE MS Spectrum

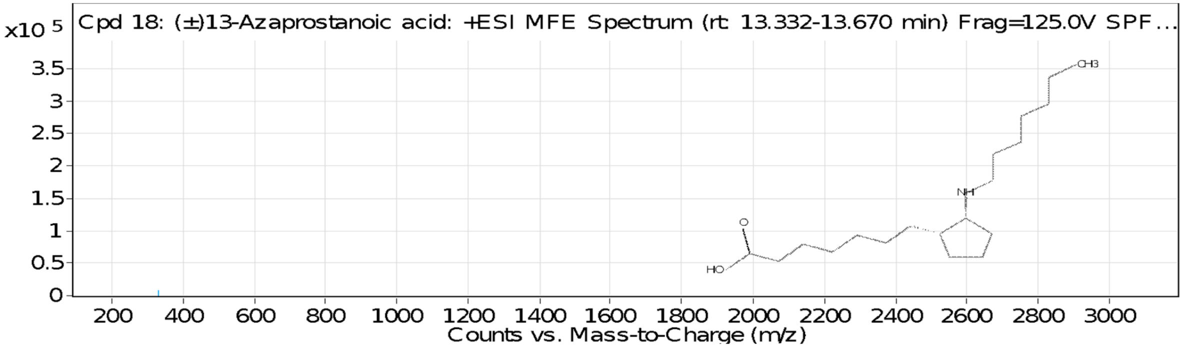

<u>Compound ix: Estra-1,3,5(10)-triene-2,17beta-diol</u>

Compound Label	Name	*m/z*	RT	Algorithm	Mass
Cpd 19: Estra-1,3,5(10)-triene-2,17beta-diol	**Estra-1,3,5(10)-triene-2,17beta-diol**	273.1848	14.323	Find by Molecular Feature	272.1774

Compound Chromatograms

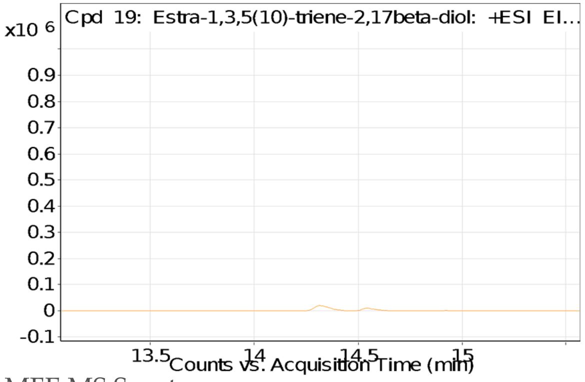

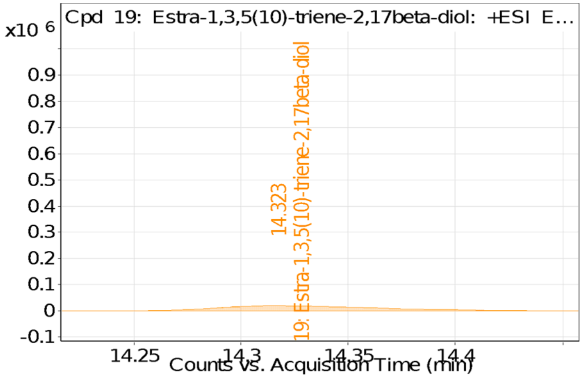

MFE MS Spectrum

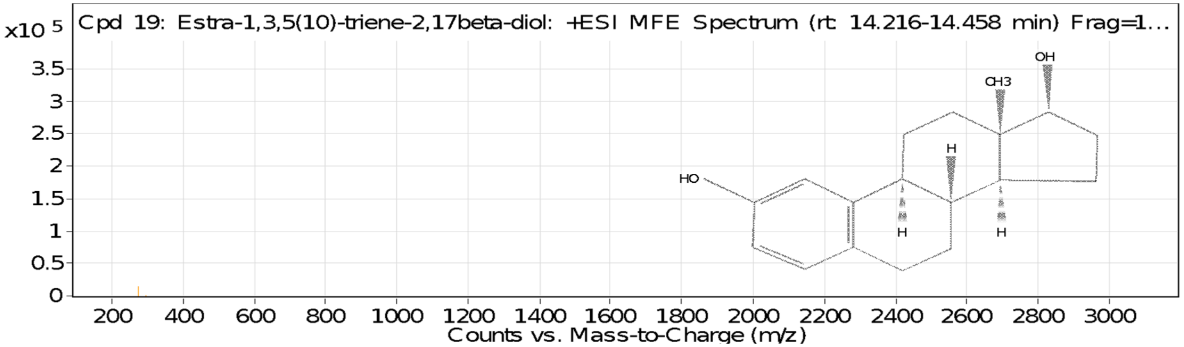

Compound x: 15(S)-15-methyl PGF2α ethyl amide

**Compound Label**	**Name**	***m/z***	**RT**	**Algorithm**	**Mass**
Cpd 22: 15(S)-15-methyl PGF2α ethyl amide	**15(S)-15-methyl PGF2α ethyl amide**	396.3106	16.521	Find by Molecular Feature	395.3029

Compound Chromatograms

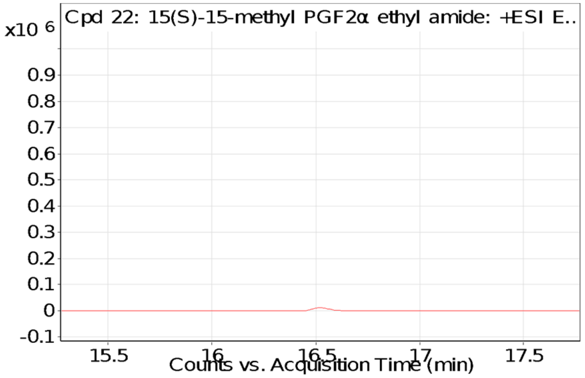

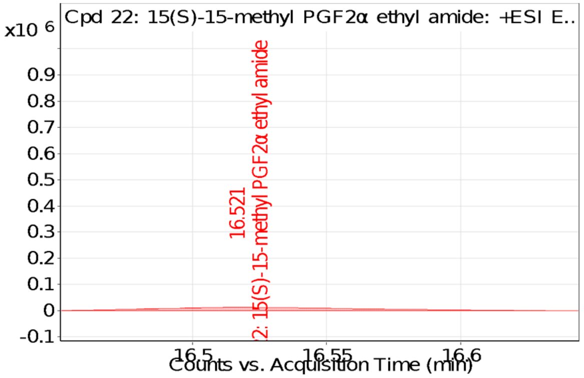

MFE MS Spectrum

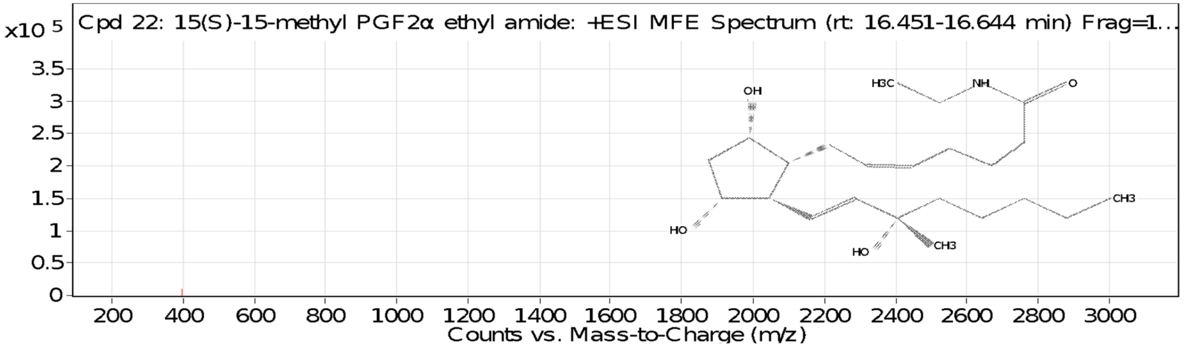

Compound xi: Emmotin A

Compound Label	Name	*m/z*	RT	Algorithm	Mass
Cpd 23: Emmotin A	**Emmotin A**	279.1594	16.743	Find by Molecular Feature	278.1521

Compound Chromatograms

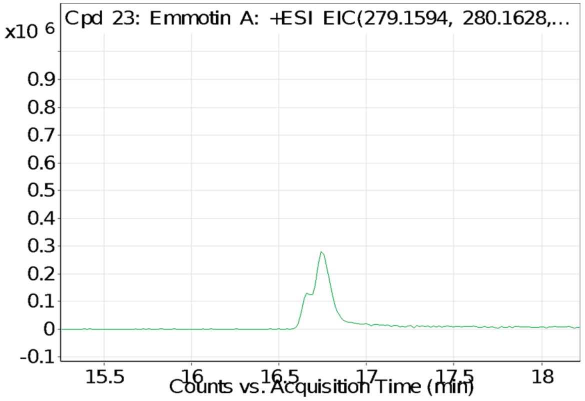

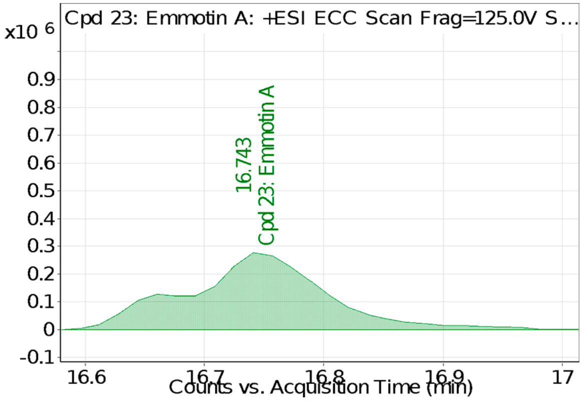

MFE MS Spectrum

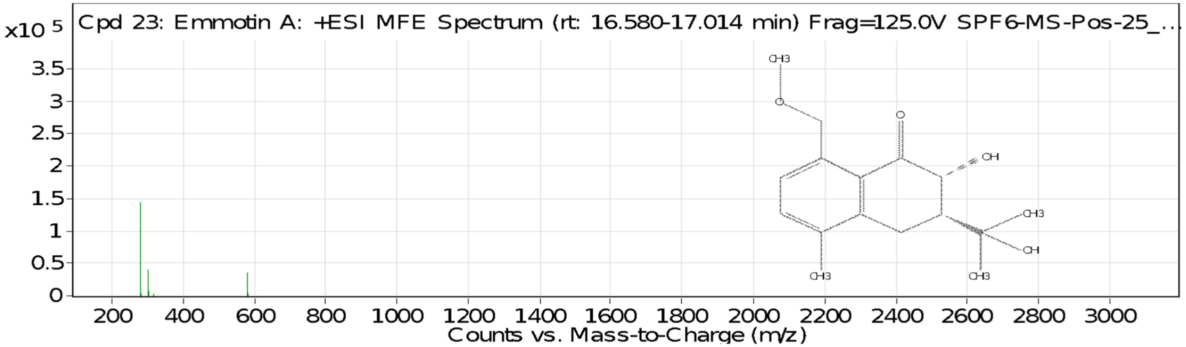

Compound xii: **3-Butylidene-7-hydroxyphthalide**

**Compound Label**	**Name**	***m/z***	**RT**	**Algorithm**	**Mass**
Cpd 25: 3-Butylidene-7-hydroxyphthalide	**3-Butylidene-7-hydroxyphthalide**	205.0858	16.755	Find by Molecular Feature	204.0786

Compound Chromatograms

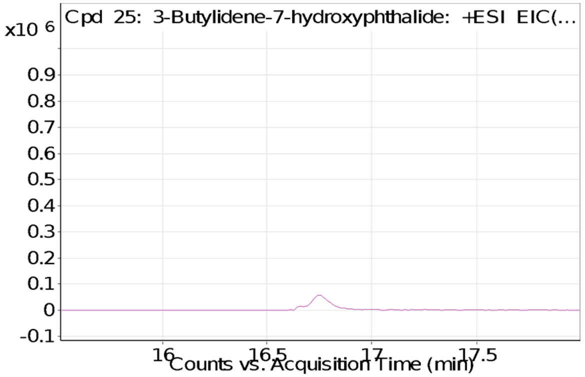

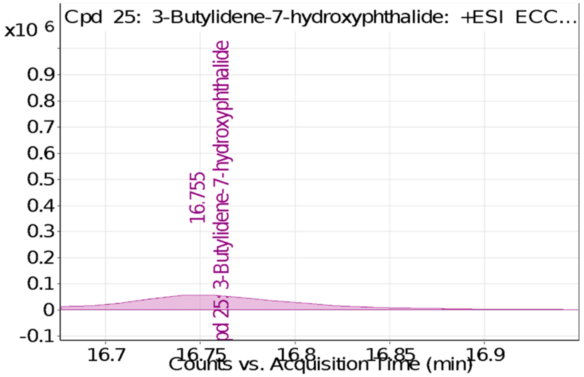

MFE MS Spectrum

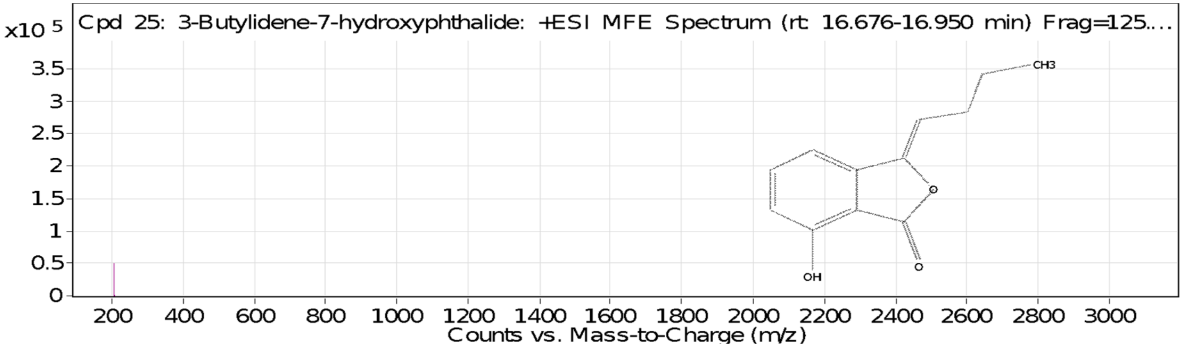

Compound xiii: N-cis-tetradec-9Z-enoyl-L-Homoserine lactone

**Compound Label**	**Name**	***m/z***	**RT**	**Algorithm**	**Mass**
Cpd 26: N-cis-tetradec-9Z-enoyl-L-Homoserine lactone	**N-cis-tetradec-9Z-enoyl-L-Homoserine lactone**	310.2374	16.922	Find by Molecular Feature	309.2303

Compound Chromatograms

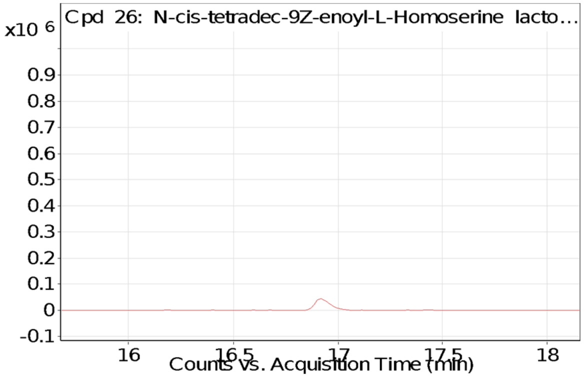

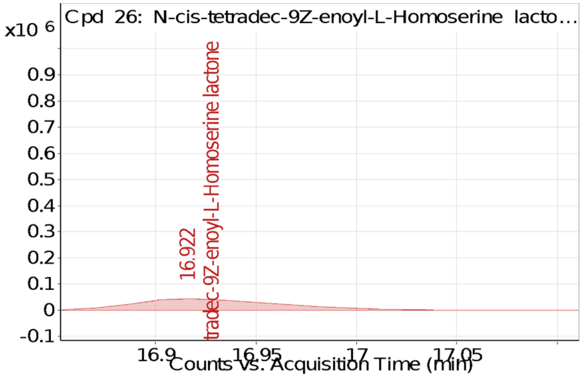

MFE MS Spectrum

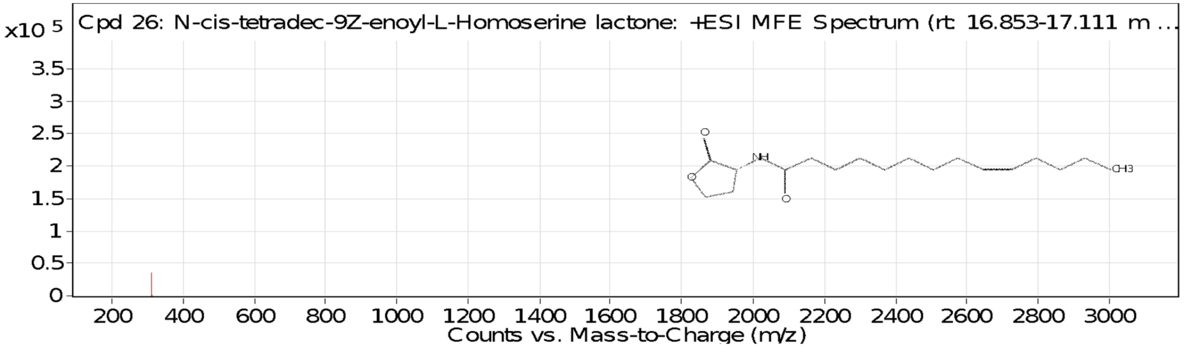

Compound xiv: Stigmatellin Y

Compound Label	Name	*m/z*	RT	Algorithm	Mass
Cpd 28: Stigmatellin Y	**Stigmatellin Y**	502.3166	17.944	Find by Molecular Feature	484.2826

Compound Chromatograms

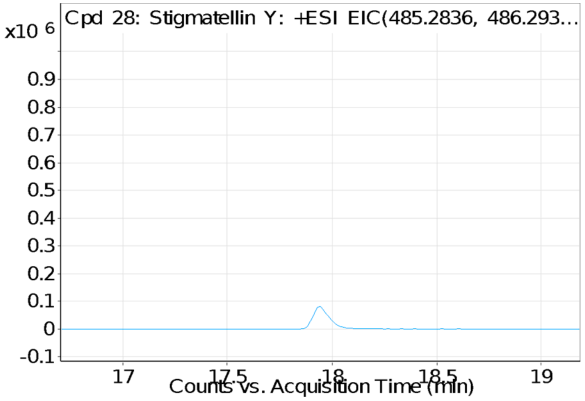

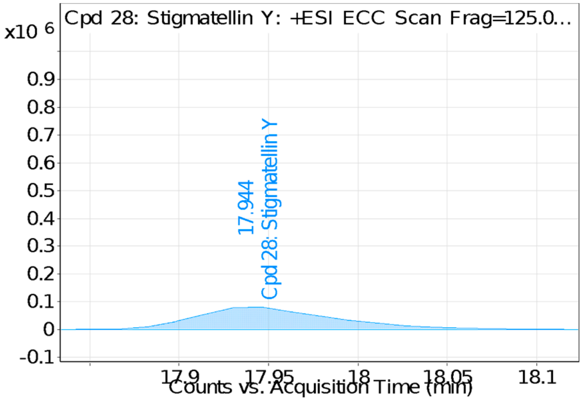

MFE MS Spectrum

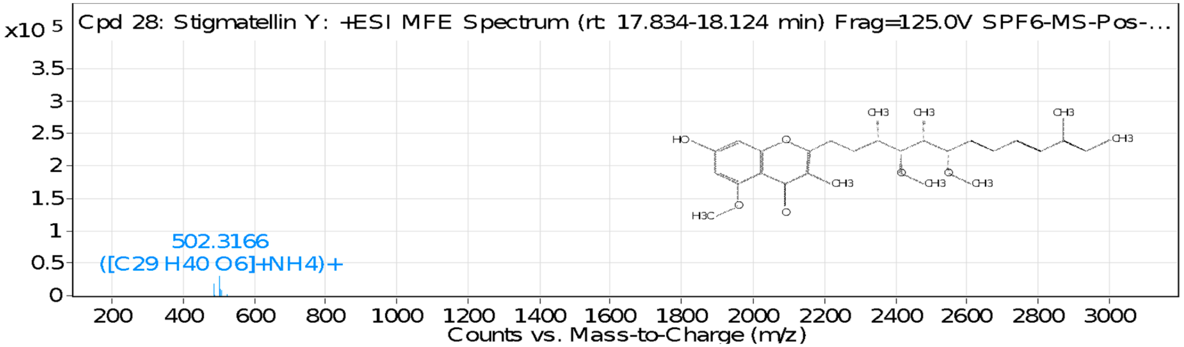

Compound xv: **Palmitic amide**

Compound Label	Name	*m/z*	RT	Algorithm	Mass
Cpd 33: Palmitic amide	**Palmitic amide**	256.2636	19.103	Find by Molecular Feature	255.2562

Compound Chromatograms

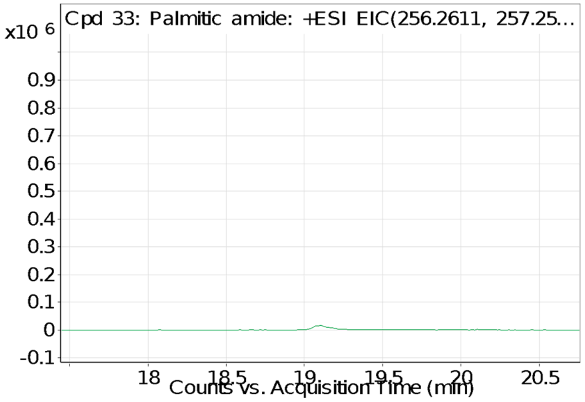

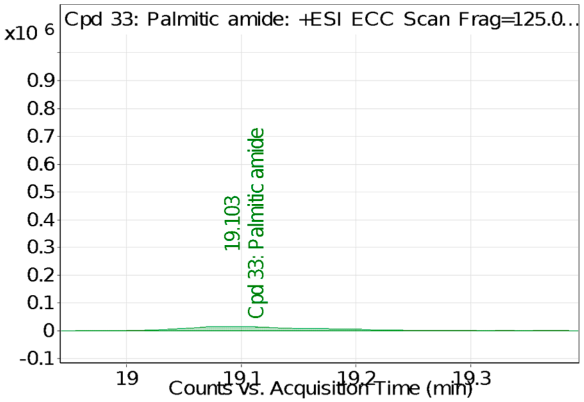

MFE MS Spectrum

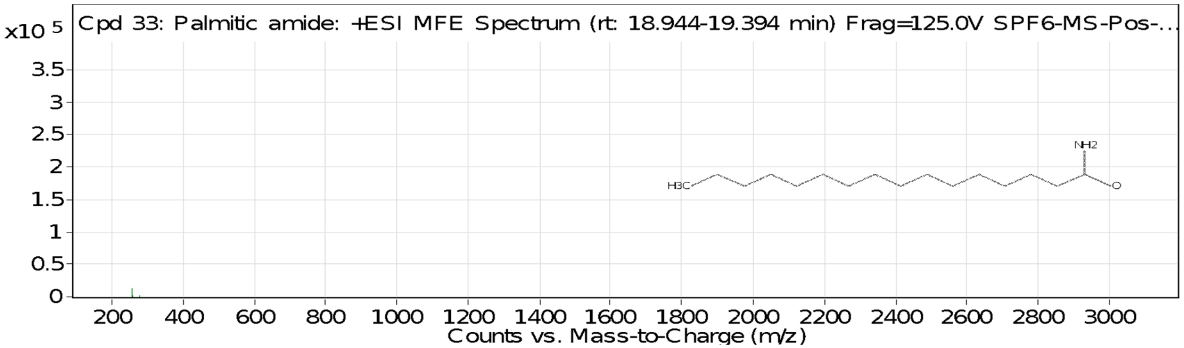

Compound xvi: 1-monopalmitin

Compound Label	Name	*m/z*	RT	Algorithm	Mass
Cpd 35: 1-monopalmitin	**1-monopalmitin**	353.2669	19.299	Find by Molecular Feature	330.2773

Compound Chromatograms

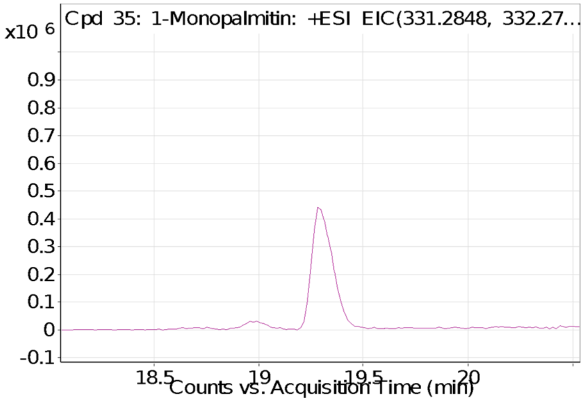

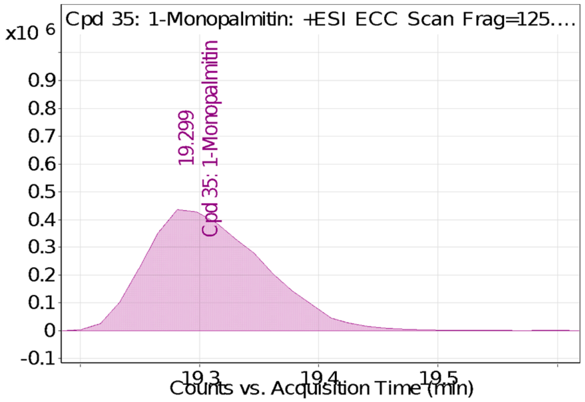

MFE MS Spectrum

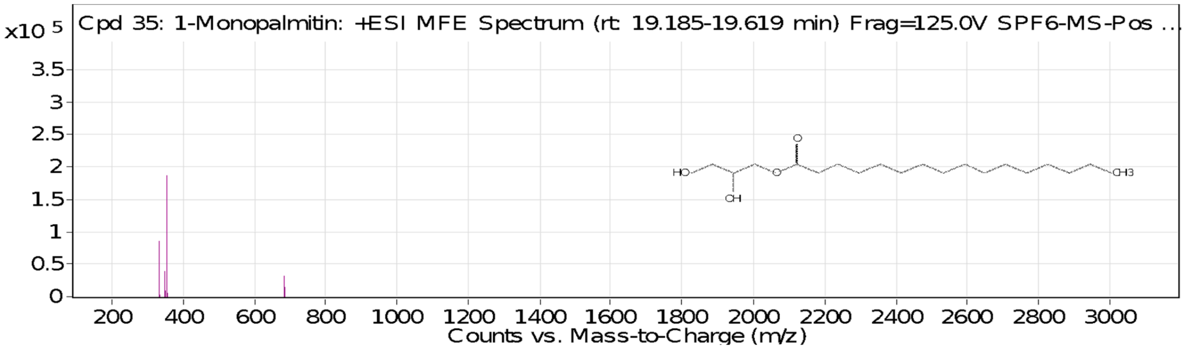

Compound xvii: Harderoporphyrin

Compound Label	Name	*m/z*	RT	Algorithm	Mass
Cpd 38: Harderoporphyrin	**Harderoporphyrin**	609.2708	19.495	Find by Molecular Feature	608.2636

Compound Chromatograms

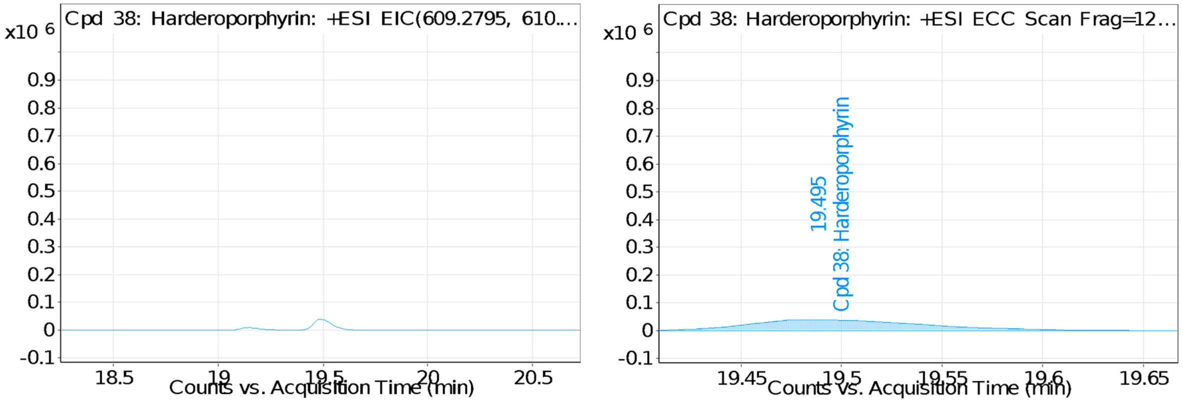

MFE MS Spectrum

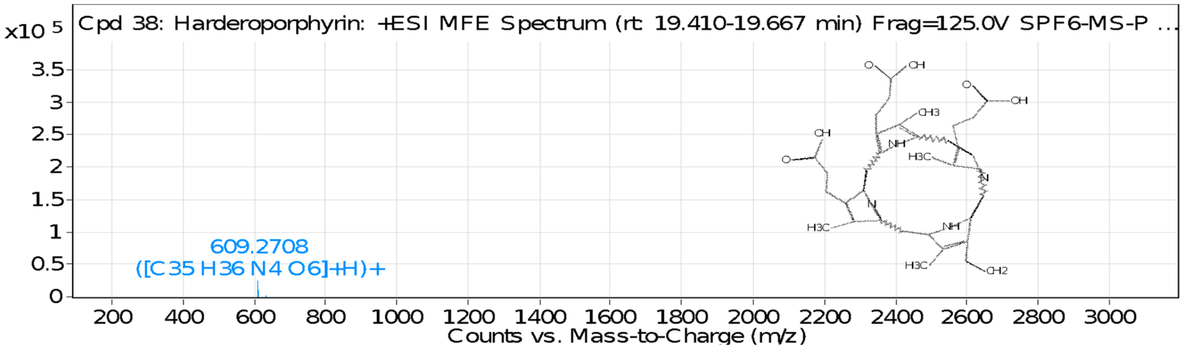

Compound xviii: Hexadecyl acetyl glycerol

Compound Label	Name	*m/z*	RT	Algorithm	Mass
Cpd 46: Hexadecyl acetyl glycerol	**Hexadecyl acetyl glycerol**	381.2983	20.782	Find by Molecular Feature	358.3092

Compound Chromatograms

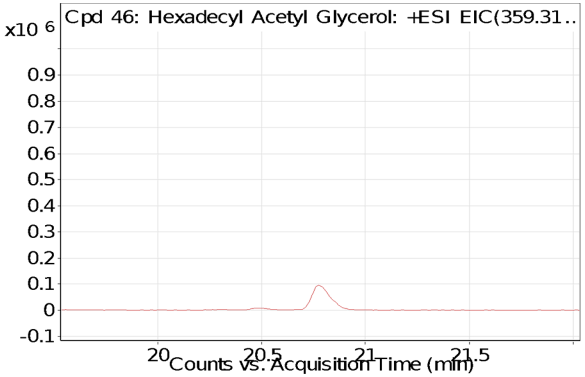

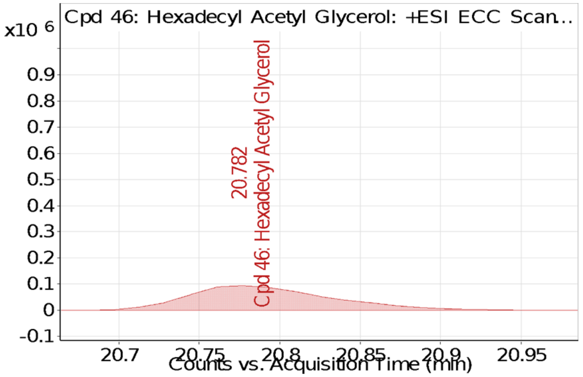

MFE MS Spectrum

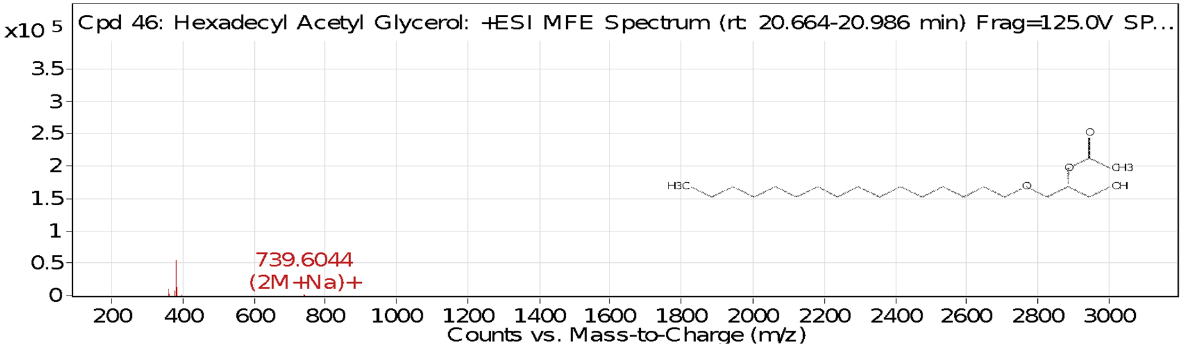

Compound xix: 3α,12α-Dihydroxy-5β-chol-8(14)-en-24-oic acid

Compound Label	Name	*m/z*	RT	Algorithm	Mass
Cpd 49: 3α,12α-Dihydroxy-5β-chol-8(14)-en-24-oic acid	**3α,12α-Dihydroxy-5β-chol-8(14)-en-24-oic acid**	391.2854	21.109	Find by Molecular Feature	390.278

Compound Chromatograms

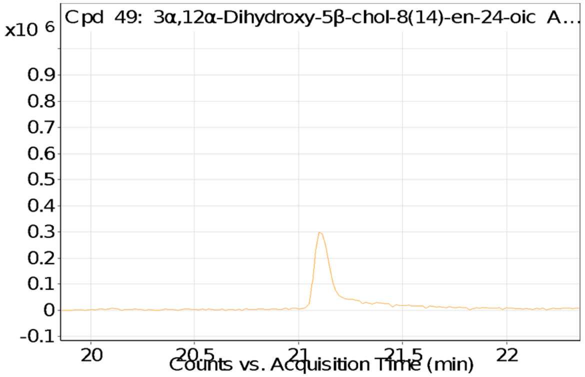

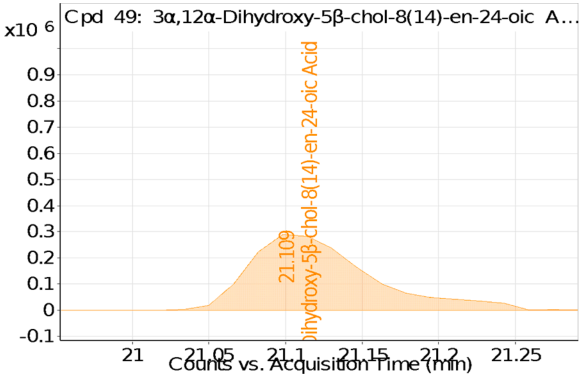

MFE MS Spectrum

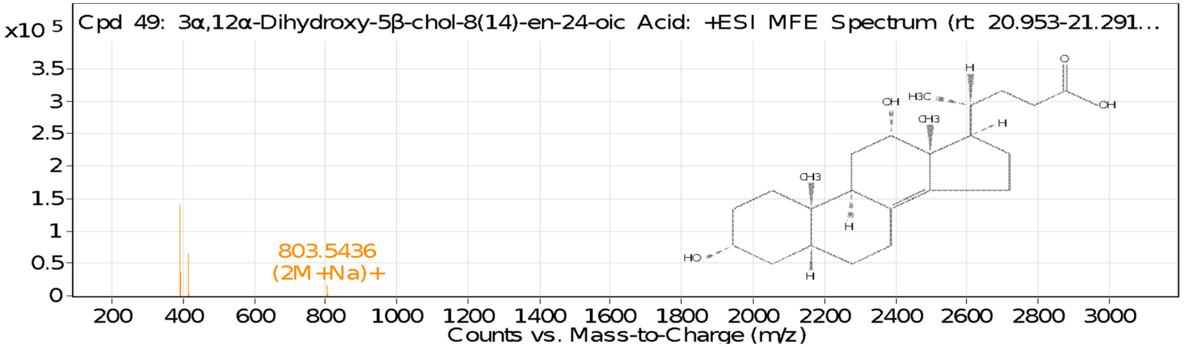

Compound xx: Docosanedioic acid

Compound Label	Name	*m/z*	RT	Algorithm	Mass
Cpd 51: Docosanedioic acid	**Docosanedioic acid**	371.3151	21.202	Find by Molecular Feature	370.3077

Compound Chromatograms

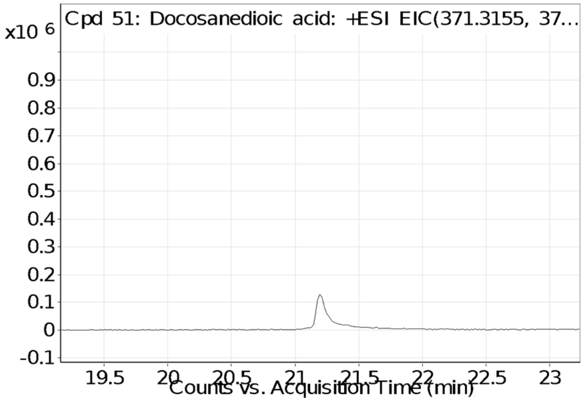

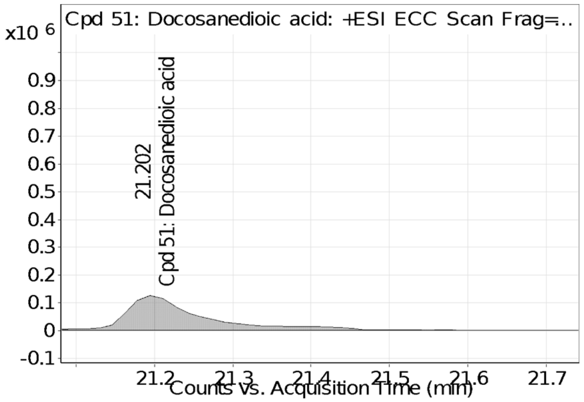

MFE MS Spectrum

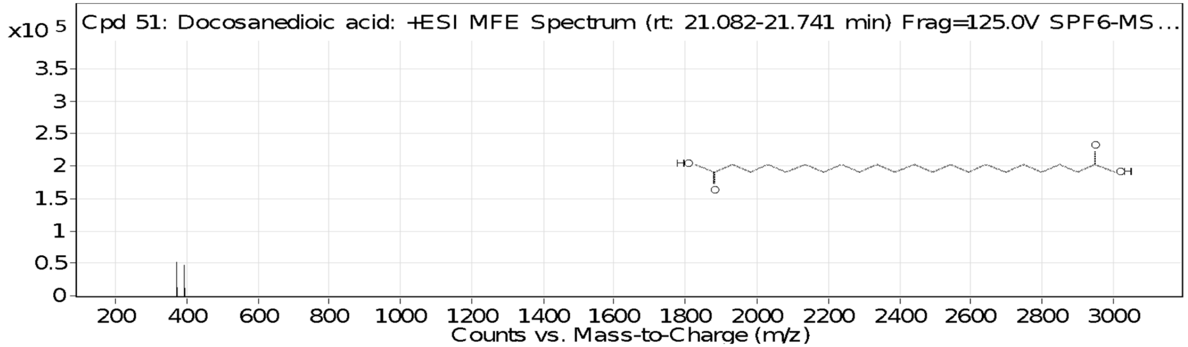

Compound xxi: Hexacosanedioic acid

Compound Label	Name	*m/z*	RT	Algorithm	Mass
Cpd 58: Hexacosanedioic acid	**Hexacosanedioic acid**	449.3598	23.533	Find by Molecular Feature	426.3705

Compound Chromatograms

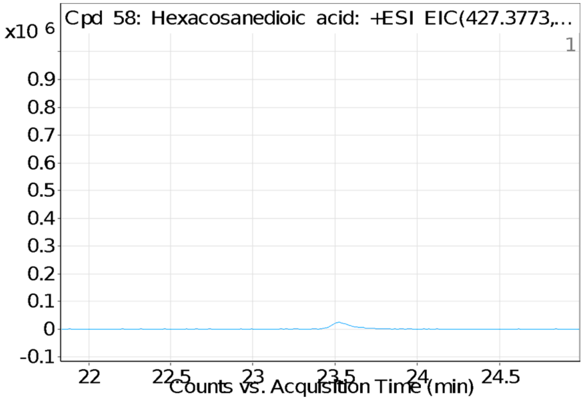

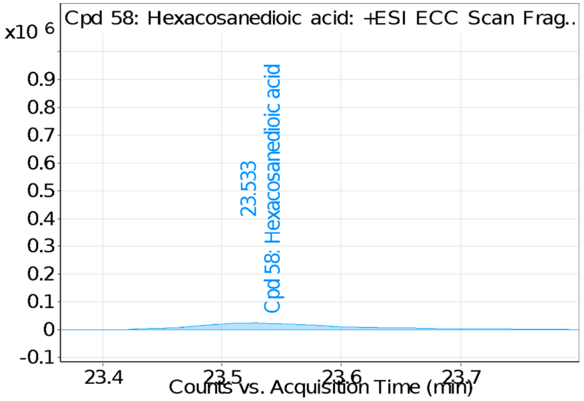

MFE MS Spectrum

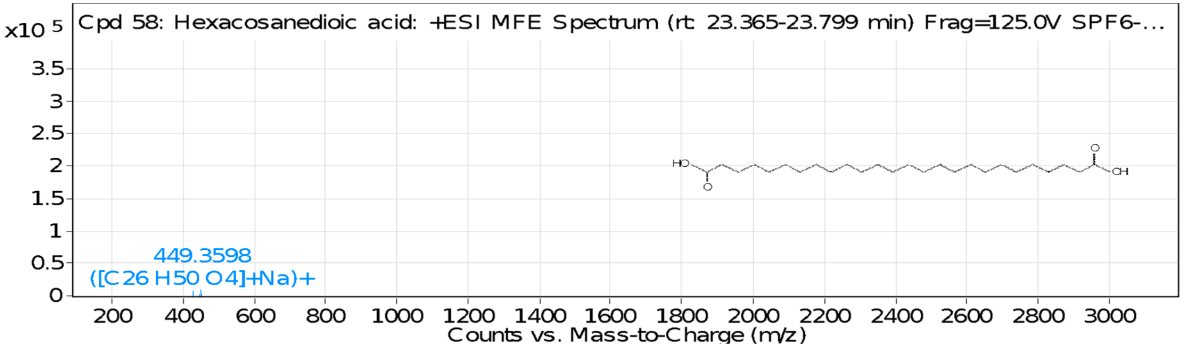

Supplement: Supplementary file 1 [file metabolites-12-01147-s001.zip › metabolites-2019640-supplementary.pdf]
